# High-Content Analysis of CRISPR-Cas9 Gene-Edited Human Embryonic Stem Cells

**DOI:** 10.1016/j.stemcr.2015.11.014

**Published:** 2016-01-12

**Authors:** Jared Carlson-Stevermer, Madelyn Goedland, Benjamin Steyer, Arezoo Movaghar, Meng Lou, Lucille Kohlenberg, Ryan Prestil, Krishanu Saha

**Affiliations:** 1 Wisconsin Institute for Discovery, University of Wisconsin-Madison, Madison, WI 53715, USA; 2 Department of Biomedical Engineering, University of Wisconsin-Madison, Madison, WI 53715, USA; 3 Department of Medical History and Bioethics, University of Wisconsin-Madison, Madison, WI 53715, USA

## Abstract

CRISPR-Cas9 gene editing of human cells and tissues holds much promise to advance medicine and biology, but standard editing methods require weeks to months of reagent preparation and selection where much or all of the initial edited samples are destroyed during analysis. ArrayEdit, a simple approach utilizing surface-modified multiwell plates containing one-pot transcribed single-guide RNAs, separates thousands of edited cell populations for automated, live, high-content imaging and analysis. The approach lowers the time and cost of gene editing and produces edited human embryonic stem cells at high efficiencies. Edited genes can be expressed in both pluripotent stem cells and differentiated cells. This preclinical platform adds important capabilities to observe editing and selection in situ within complex structures generated by human cells, ultimately enabling optical and other molecular perturbations in the editing workflow that could refine the specificity and versatility of gene editing.

## Highlights

• High-content analysis of arrayed hESC colonies increased gene-editing efficiency
• Rapid one-pot transcription of sgRNAs can be multiplexed to edit hESCs
• hESCs gene edited on ArrayEdit exhibited proper phenotypes
• ArrayEdit provides a new window into the process of gene editing human cells

## Graphical Abstract

**Figure:**
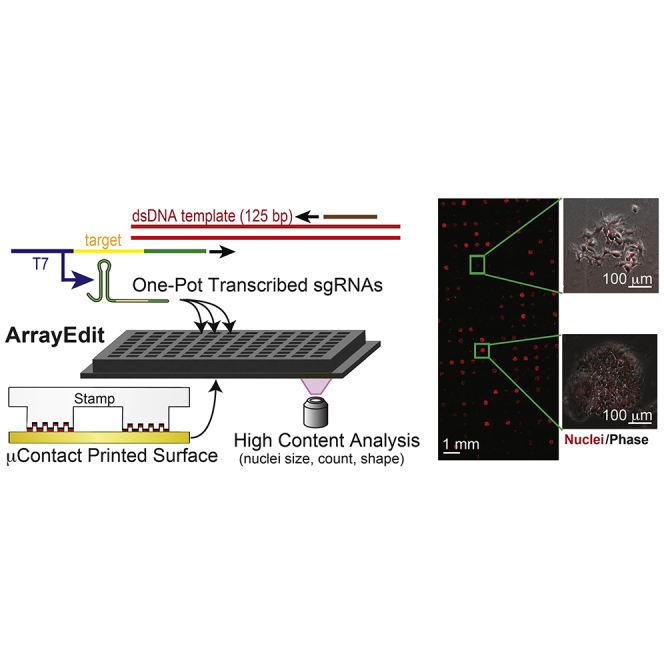

## Teaser

Saha and colleagues show that a new culture and imaging platform, called ArrayEdit, allows both high-throughput and high-resolution subcellular imaging of thousands of hESC colonies in parallel. ArrayEdit generated CRISPR-Cas9-edited, living hESC lines at 82% efficiency within days. Biallelic edited cell lines at the *LAMA5* locus did not contain any detectable off-target mutations and exhibited expected self-renewal defects upon subsequent characterization.

## Introduction

CRISPR-Cas9, an emerging genome surgery tool, exploits an engineered ribonucleoprotein complex consisting of two essential components: (1) a protein, Cas9; and (2) a single-guide RNA (sgRNA). Together, the Cas9-sgRNA complex cuts a specific target sequence in the genome. Human cells and tissues edited by CRISPR-Cas9 are important resources for drug target identification (Kasap et al., 2014, Shi et al., 2015, Smurnyy et al., 2014), regulatory science (Hsu et al., 2014), medicine (Doudna, 2015), and basic biology (Hsu et al., 2014, Sternberg and Doudna, 2015). However, human gene-editing experiments frequently require laborious cloning of expression plasmids for each sgRNA, and there are limited opportunities in these culture systems to watch and perturb genome surgery in action, as it is difficult to isolate and image living mutant cells during and shortly after the DNA cleavage event. Overall, there is a need to expand the throughput and capabilities of current in vitro human culture systems where novel genome surgery approaches can be evaluated with human cells and tissues (Baltimore et al., 2015). Advanced capabilities with human pluripotent stem cells in particular could eventually expand the suite of human preclinical model systems, ranging from patient-specific cell lines to complex human embryonic tissues established from stem cells.

Current gene-editing techniques generate heterogeneous human cell populations that require significant subsequent characterization. It is crucial to analyze the genome of the edited cells by sequencing before continuing with other studies, and several protocols require destruction of mutant cell populations during sequencing analysis (Ding et al., 2013, Kasap et al., 2014, Mali et al., 2013, Miyaoka et al., 2014, Shi et al., 2015, Smurnyy et al., 2014, Yang et al., 2013). For example, targeted gene disruption followed by selection and next-generation sequencing can identify drug targets, but a separate, subsequent gene-editing experiment is required to obtain living mutant cells for downstream analysis (Kasap et al., 2014, Sanjana et al., 2014, Shalem et al., 2014, Shi et al., 2015, Smurnyy et al., 2014, Wang et al., 2014), a process that is often infeasible for slowly dividing or primary cells. This slows epigenomic and functional characterization of properly edited cells, and it is currently unknown whether there are persistent epigenomic and functional problems within the edited cells (Bosley et al., 2015). Further sequence-level characterization is also required at the single clone level, as there is frequent and variable disruption of, or insertion of donor DNA into, the non-targeted allele in edited cell lines (Merkle et al., 2015). Finally, efficiencies of isolating precisely edited cells remain a challenge with current methods, typically with 20% or lower efficiencies to make near-precise deletions in the human genome (Byrne et al., 2015).

Here, we describe a platform, termed ArrayEdit, that combines two capabilities: one-pot transcription, and the combination of microcontact printed plates and high content analysis (HCA). First, we describe a method that can generate many sgRNAs in parallel, within hours, using chemically synthesized oligonucleotides ordered in a multiwell format. One-pot transcribed sgRNAs can be delivered without purification and can efficiently generate desired gene edits within human embryonic stem cells (hESCs) when co-delivered with Cas9. Second, we describe a versatile combination of culture and imaging to select edited cells and tissues using non-destructive analysis of thousands of spatially defined features that localize edited cell colonies/aggregates. We were able to isolate gene-edited hESC lines within 2 weeks, 82% of which were mutant for our desired edit at a proof-of-concept locus (*LAMA5)* without any detectable off-target mutations. This platform adds important capabilities to easily observe editing and selection in situ within complex structures generated by human cells.

## Results

### Simplified One-Pot Transcription of sgRNAs in Multiwell Plates

**Figure 1:**
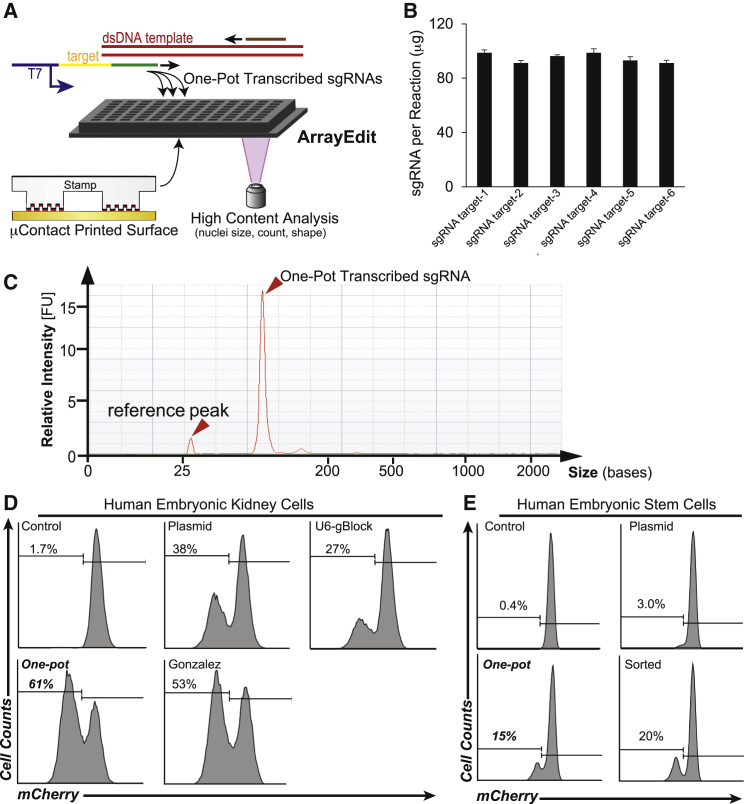
ArrayEdit: an Arrayed, High-Content Platform to Monitor and Isolate Gene-Edited Cells via One-Pot Transcribed Single-Guide RNAs (A) Overview of ArrayEdit assembly and key components. Top: Schematic of one-pot PCR and T7 transcription. All components can be mixed and reacted within a single well without any intermediate purification steps. Primers are synthesized as custom oligonucleotides. The forward primer defines the genomic target of editing by Cas9. Bottom: Surface modification to the bottom of multiwell plates generates cell-adhesive μFeatures on a glass bottom. Each μFeature can be tracked over time via high-content imaging and stitched together to form a time-lapse visualization of edited cell phenotypes. (B) Amount of sgRNA produced within each well via one-pot transcription. Data are represented as means ± 95% CI from four independent one-pot transcriptions on each sgRNA target (targets 1–3, *mCherry-1-3*; 4–6, *GFP-1*-*3*) and are not significantly different (Student's t-test, *p* > 0.05, Bonferroni correction). (C) RNA Bioanalyzer spectra of one-pot transcribed sgRNA. Narrow peak (arrowhead) is consistent with only the desired product being produced. The reference peak is used by the Bioanalyzer to standardize size measurements. (D and E) Flow cytometry histograms of HEK-H2B-mCherry cells (D) and WA09-H2B-mCherry hESCs (E) 4 days after delivery of *mCherry-1* sgRNAs. sgRNA was either expressed of a plasmid, ordered commercially (U6-gBlock), created using previously described methods (Gonzalez), or one-pot transcribed (n = 2; independent experiments). Sorted: a GFP plasmid is co-electroporated and then sorted for GFP+ cells, leading to enrichment of cells that contained exogenous nucleic acids. See also Figure S1 and Tables S1 and S2.

One key feature of ArrayEdit is the generation of one-pot transcribed sgRNAs with chemically synthesized oligonucleotides within a multiwell format. One-pot transcription is similar to one-pot synthesis in chemistry, because products of the reaction are created at high yields without any intermediate purification steps. As outlined in Figure 1A, our method consists of three components: (1) a forward primer containing a minimal T7 primer, sgRNA target sequence, and a region for PCR amplification; (2) a double-stranded sequence of DNA encoding the sgRNA conserved region; and (3) a universal reverse primer for PCR amplification (see Figure S1A and Tables S1 and S2 for sequences). This method is versatile and can generate any desired sgRNA within hours, regardless of sequence complexity. In contrast to other methods (González et al., 2014, Liang et al., 2015, Lin et al., 2014), this process is modular, such that advances in the sgRNA backbone that refine specificity or increase editing efficiency (Chen et al., 2013, Shechner et al., 2015) do not necessitate recreating entire sgRNA libraries. All primers can be chemically synthesized and delivered from commercial vendors overnight, decreasing the time between design and experiments.

To demonstrate the multiplexed synthesis of sgRNAs, we designed six sgRNAs targeting two genes (*mCherry*, *GFP;* Table S2) and ordered four replicates of each primer set in a 96-well format for subsequent one-pot transcription. Next, PCR amplification was performed yielding a DNA product that was of consistent size and concentration across all targets (Figure S1B). These DNA products are reusable and can be stored for months, or used immediately for in vitro transcription (IVT) with T7 RNA polymerase. IVT was allowed to proceed for as little as 2 hr to overnight. Longer incubation times resulted in increased concentration without any undesired products or degradation of the sgRNA. When allowed to progress overnight, IVT consistently produced greater than 90 μg of sgRNA in a 20-μl reaction (Figure 1B), suggesting that the concentration of rNTPs is the limiting reagent. With consistent yields, this strategy renders post-production purification or quantification unnecessary and allows us to use approximate molar quantities of sgRNA during transfection directly following IVT. This method of production also reliably produces only the desired product of the correct size as seen in a lone narrow peak when profiled by an RNA Bioanalyzer 2100 (Figure 1C).

We designed and created one-pot sgRNAs to target 74 additional loci (Table S2) in both hESCs and human embryonic kidney (HEK) cells. After transfecting these sgRNAs into cells, we determined the percent gene modification in an *EGFP* transgene via flow cytometry (Figure S1C) and in endogenous *FGFR2* via restriction fragment-length polymorphism on genomic DNA (Figure S1D). Further, we detected large-scale genome deletions in *MUC16* and *DPH7* using gel electrophoresis on genomic DNA (Figures S1E and S1F). Large-scale genome deletions were created by transfecting cells with two sgRNAs that are located 200–4,000 bp apart from each other in the genome. A standard agarose gel was then able to resolve if the sequence between the two sgRNAs was deleted based on amplicon size. In all cases, each one-pot sgRNA was capable of creating a targeted DNA double-strand break that was likely resolved using non-homologous end-joining (NHEJ).

Next, we compared the gene-editing efficiency of one-pot transcribed sgRNAs against the established methods of sgRNA production. To quantify single-cell editing efficiency, we used flow cytometry in conjunction with a transgenic HEK-H2B-mCherry line, which was engineered to constitutively express a fusion protein of histone 2B (H2B) and mCherry from a single *AAVS1* safe-harbor locus (Figure S1G). sgRNAs were produced via one-pot transcription, plasmid transfection (Mali et al., 2013), commercially (termed a U6-gBlock), and via previously described IVT methods (González et al., 2014). One-pot transcribed sgRNAs resulted in the highest percentage of fluorescence expression loss, successfully knocking out expression in 61% of cells (Figures 1D and 1E). In addition, one-pot sgRNA production was the quickest method, requiring only 2 days from design to experiment, whereas commercially produced sgRNAs required 4–5 business days and previously described methods required a cloning step and plasmid production scale-up (∼4 days). We then used one-pot transcribed sgRNAs with a transgenic hESC line: WA09-H2B-mCherry (Harkness et al., 2015). When one-pot transcribed sgRNAs targeting *mCherry* were introduced via electroporation into this line, some hESCs lost mCherry expression after 4 days of culture. In addition, one-pot transcribed sgRNAs generated five times more mCherry-negative cells when analyzed by flow cytometry than standard techniques that express the same sgRNAs from plasmids (Mali et al., 2013) (Figure 1E). One-pot transcribed sgRNAs also performed nearly as well as methods that enrich for transfected cells (Ding et al., 2013) via fluorescence-activated cell sorting (FACS) for GFP in a co-transfection with a GFP-expressing plasmid.

### Deep Sequencing of Edited Stem Cell Derivatives

**Figure 2:**
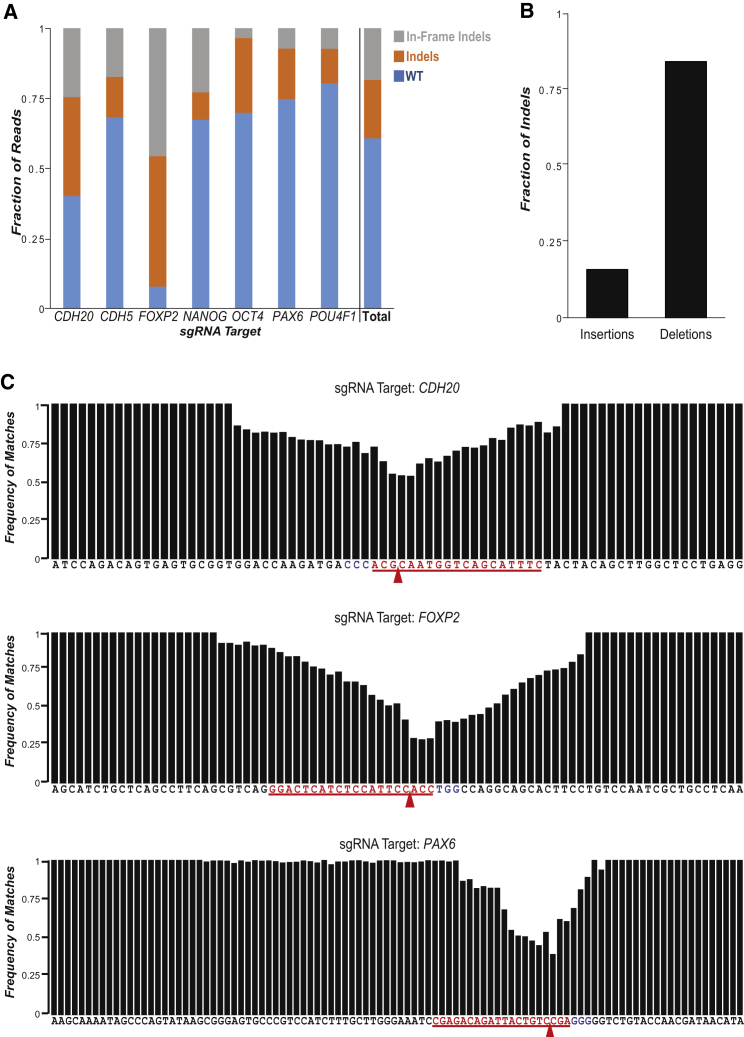
Deep Sequencing Reveals High-Efficiency Modification by One-Pot Transcribed sgRNAs (A) Fraction of sequencing reads from gene-edited, hESC-derived embryoid bodies that matched the wild-type (WT) sequence (blue), contained at least one insertion or deletion (indel, orange), or contained an in-frame indel (gray). Edits occurred in genes and transcripts marking all three germs layers (ectoderm [*PAX6*, *POU4F1*], mesoderm [*CDH5*], endoderm [*CDH20*, *FOXP*2]) and pluripotent stem cells (*NANOG*, *OCT*4). Total: all reads across the seven loci. (B) Frequency of insertion events to deletion events in the sequencing reads, with deletion being ∼5× more common than insertion. (C) Per base frequency of matches to the wild-type sequence for three genes (*CDH20, FOXP2, PAX6*). Red bases denote the sgRNA target sequence while blue bases denote the protospacer adjacent motif (PAM). Red arrows indicate the predicted site of double-strand break formation by Cas9. The beginning and ending sequences (17–22 bp) for each gene are uniform, because they contain reads that were amplified using primers (17–22 bp) during PCR.

To gain a more detailed analysis of genome-editing events in hESCs and their matured cell derivatives, we performed deep sequencing of cells edited by one-pot transcribed sgRNAs. We first generated one-pot transcribed sgRNAs targeting seven genes that mark pluripotent, ectodermal, mesodermal, and endodermal cells. These one-pot sgRNAs were electroporated into a HUES8 hESC line with an inducible Cas9 transgene (González et al., 2014). The electroporated hESCs were cultured for several passages and then matured into embryoid bodies (EBs) for 5 days to allow cells to differentiate into all three germ layers. mRNA was extracted from the EBs and reverse transcribed into cDNA. PCR using primers flanking each of the sgRNA target sites was performed and prepared for sequencing via Illumina Hi-Seq. We found 20%–92% of reads overlapping the target sites contained at least one insertion or deletion (indel) within a ∼100-nucleotide window around the expected cut site (Figure 2A). Consistent with NHEJ repair at the expected cut site, both frameshift and in-frame indels were observed for all of these loci (Figure 2A, gray, orange). We also observed that many more reads with an indel contained a deletion event (85%) than contained an insertion event (15%) (Figure 2B). Similar observations were reported in deep sequencing analysis of human cells edited by *S. pyogenes* Cas9 (Mali et al., 2013, Schumann et al., 2015).

The sequencing results allowed quantitative analysis of observed indel mutations and their spatial distribution in the target region. The results in Figure 2C show the frequency of indels in the endodermal markers, *CDH20* and *FOXP2*, and an ectodermal marker, *PAX6*. In EBs derived from edited cells, we found the highest frequency of indels three to four nucleotides upstream from the protospacer adjacent motif (PAM) sequence (Figure 2C), consistent with reports of type II CRISPR systems. Taken together, one-pot sgRNAs, when combined with Cas9, can generate targeted genomic edits in hESCs that can be expressed in differentiated cells.

### Patterning Adhesive Microfeatures to Separate Gene-Edited hESCs

In previous experiments, all gene-edited cells within standard cell culture were interspersed with wild-type cells, so colonies would need to be selected, dissociated, and subcloned to isolate gene-edited cells for subsequent culture and analysis. Such a mixture can be easily visualized in the *mCherry*-edited cells (Figure S1H). To overcome laborious downstream clonal selection steps in the editing workflow, we designed our ArrayEdit platform to separate edited cells by exploiting microcontact printing (μCP) on the surface of multiwell plates. μCP was performed on gold-coated glass (Harkness et al., 2015) to create surfaces within standard culture multiwell plates that contained greater than 400 circular μFeatures of 300 μm diameter per well, allowing for the spatially controlled growth of up to 2,400 separate gene-edited cells per standard 6-well tissue culture plate (Figure S2A). The poly(ethylene glycol) (PEG) brush surface layer after μCP does not contain defects common to other stamped PEG surfaces, and μFeatures are stable upon extended culture for over 30 days (Sha et al., 2013).

**Figure 3:**
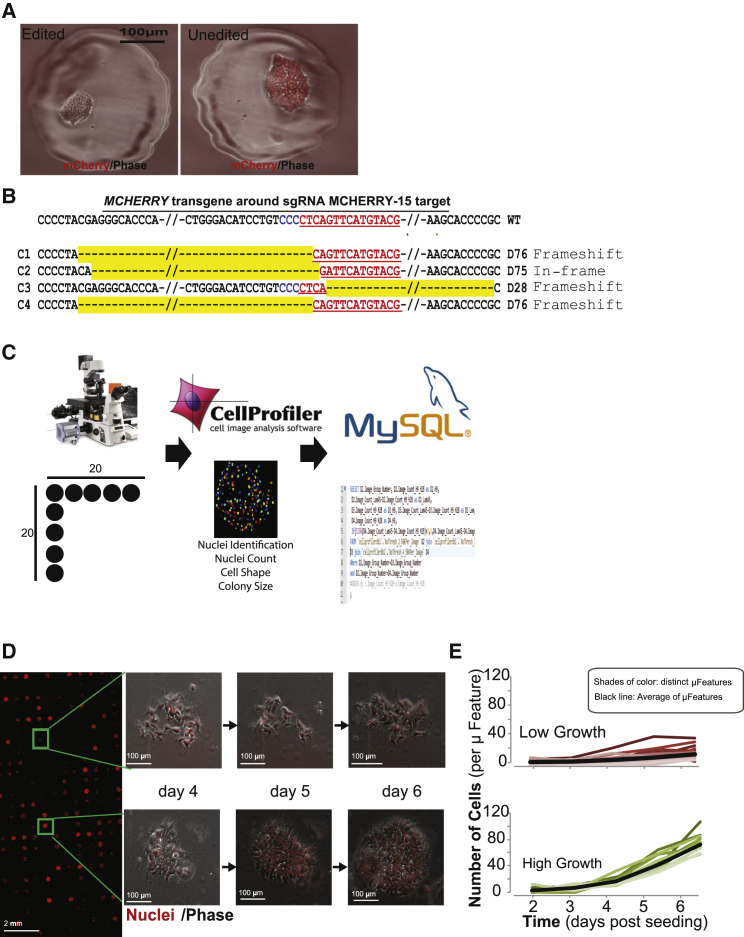
Substrate Micropatterning Enables Live HCA of Gene Editing (A) Isolation of homogeneous *mCherry* gene-edited hESC lines on micropatterned plates. Clonal knockouts can be reliably identified and expanded in spatial isolation on each μFeature. (B) Sanger sequencing of *mCherry*-edited clones isolated via ArrayEdit. The sgRNA target is denoted in red and the protospacer adjacent motif (PAM) in blue. Deletions are represented in yellow and the total length of deletion is to the right of the sequence (e.g., D76 indicates a deletion of 76 base pairs). (C) High-content image acquisition and analysis workflow. Images are taken in a 20 × 20 grid and are passed to CellProfiler. Different colors indicate distinct identified objects (nuclei). CellProfiler results are sent to a MySQL database. (D) Image of ArrayEdit within one standard culture well. Each μFeature can be tracked over time and stitched together to form a time-lapse visualization of edited cell phenotypes. Clones in two separate features are shown on days 4, 5, and 6. (E) Growth curves for cells within 24 μFeatures on ArrayEdit over 5 consecutive days after editing with LAMA5 sgRNAs. Curves were separated into high- and low-growth rate groups. See also Table S3.

ArrayEdit enabled the facile isolation of living gene-edited hESCs. We electroporated one-pot transcribed sgRNAs against *mCherry* into our WA09-H2B-mCherry labeled line along with a plasmid encoding Cas9. Cells were then seeded at clonal density on ArrayEdit according to a Poisson distribution, such that there would be high probability for 0 or 1 cell to be within each μFeature. Four days after transfection, it was trivial to identify WA09-H2B-mCherry clones of interest via fluorescent microscopy (Figure 3A). We then randomly selected four clones that lost fluorescence and transferred them to separate wells of a 24-well plate. After 5 days of subsequent culture, genomic DNA was harvested and Sanger sequenced across the expected sgRNA target site. The sequences revealed indel mutations at the desired target causing loss of mCherry fluorescence (Figure 3B). Interestingly, two of the clones isolated possessed the same modification, suggesting the presence of local DNA microhomology influencing DNA repair pathways (Bae et al., 2014).

### HCA to Identify Properly Edited hESCs

To enable marker-less identification of gene-edited cells, we developed an automated high-throughput HCA within ArrayEdit (Figure 3C). Twenty-four hours post-seeding and at each subsequent 24 hr, fluorescence microscopy was used to individually image each μFeature. Due to the array-based format and spatial control of the μFeatures, daily images can be used to create a time-lapse image of cell number within each μFeature (Figure 3D). An automated analysis pipeline to assess cell number was created in CellProfiler (Carpenter et al., 2006), a software package for image analysis. From the image, the number of nuclei per image was identified (Figure S2B) and written to a database for further analysis (Supplemental Text). Using high-throughput computing, ∼15,000 images can be analyzed in the span of a few hours. Data from individual μFeatures over multiple days were joined together to provide the growth rate for cells within each μFeature (see example in Figures 3E, S2C, and S2D).

We sought to test the speed and efficiency of ArrayEdit against prior work on *LAMA5*, because specific domains of *LAMA5* gene edits are predicted to render a selectable phenotype in several culture conditions (Laperle et al., 2015). α-5 laminin, encoded by *LAMA5*, is an extracellular matrix protein recently identified as an autocrine/paracrine factor regulating self-renewal of hESCs (Laperle et al., 2015). Isolating growth-deficient *LAMA5* mutants using standard gene editing to study the function of this factor is difficult in standard cultures, as they are quickly out competed by unwanted wild-type cells. In prior work (Laperle et al., 2015), through FACS followed by laborious subcloning of 50 gene-edited colonies, we isolated only a few lines edited at *LAMA5*.

**Figure 4:**
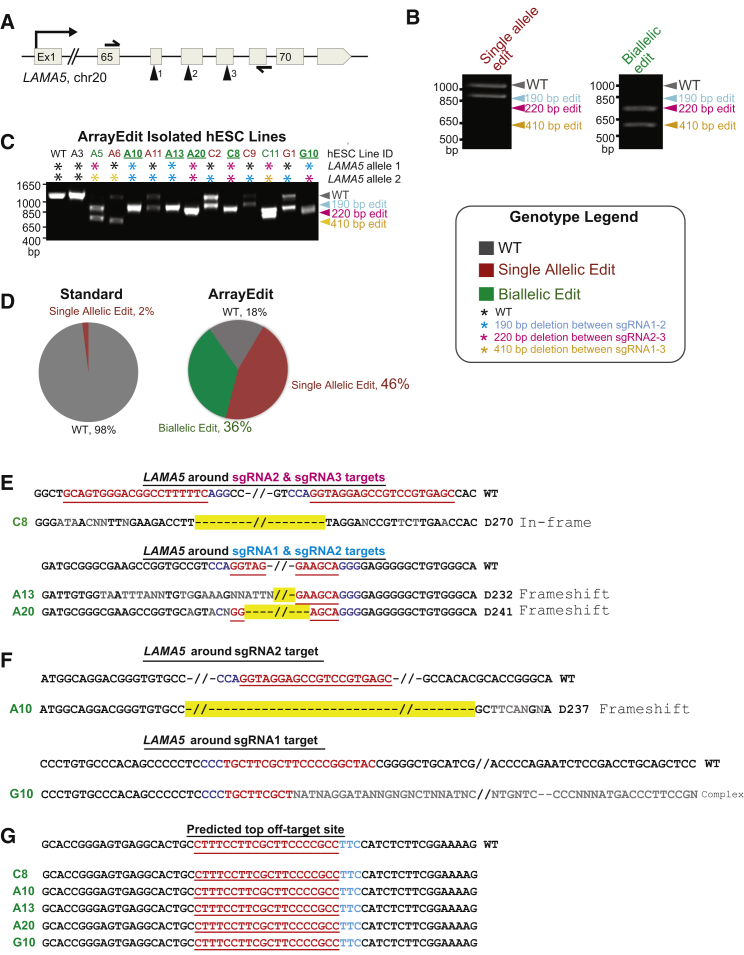
Genetic Characterization of hESC Clones Isolated from ArrayEdit (A) Schematic of CRISPR-targeted regions in *LAMA5* including the primers used for genomic amplification. (B) Agarose gel of PCR products generated from amplification of genomic DNA isolated from an ArrayEdit gene-deleted clone. Gel indicates the expected deletions spanning hundreds of base pairs. Single allele mutants can be identified by the presence of a wild-type (WT) length band, while biallelic mutants are identified by the absence of a wild-type band. (C) Agarose gel of PCR products generated from amplification of genomic DNA isolated from ArrayEdit gene-edited hESC lines. ArrayEdit was implemented on hESCs with three sgRNAs targeting *LAMA5* as shown in (A) Gel indicates the expected deletions spanning hundreds of base pairs. Red clone names denote single allele edited lines, while green denotes biallelic modifications. Each allele is denoted by a colored asterisk corresponding to which sgRNA combination is presumed to have made the modification. Underlined clones are representative clones presented in the main text. (D) Summary of genotypes obtained from hESC lines isolated after gene editing using ArrayEdit or standard procedures (see text). Efficiencies of generating edited cells are significantly higher on ArrayEdit. Genotypes are detailed in (C). (E) Sanger sequencing analysis of representative biallelic edited hESC lines chosen in (C) that displayed gene editing between two sgRNAs. Wild-type is denoted on top, and the biallelic edited hESCs are below. Color codes are the same as in Figure 3. Non-faithful nucleotides in sequence alignment are in gray and are believed to be caused by 1 or 2 bp differences in alleles, causing misreads during sequencing. (F) Sanger sequencing analysis of representative biallelic edited hESC lines chosen in (C) that displayed unexpected deletions around one sgRNA site (A10) or potential differing modifications to both alleles (G10). (G) The sequence around the top potential off-target site for Cas9 with sgRNA1 is shown at the bottom. No modifications were observed in the sequencing results from any of the edited hESC clones. See also Figures S3 and S4.

On ArrayEdit, three sgRNAs were designed that targeted consecutive exons in the globular domains of the integrin binding 3′ region of *LAMA5* (Figure 4A). One-pot transcribed sgRNAs were electroporated as a pool such that any cell that receives separate two sgRNAs can experience a deletion on the order of 100s of base pairs via NHEJ repair, which can disrupt *LAMA5* function. Exploiting HCA over a period of 6 days, we tracked 480 potential gene-edited *LAMA5* clones and observed clones that expanded rapidly as well as clones that lagged behind (Table S3, Figure 3E). Many clones followed an exponential growth model of proliferation, suggesting that they had not been edited and maintained a wild-type phenotype. However, there was another population of clones exhibiting a non-standard growth phenotype, suggesting that increased rates of apoptosis or decreased rates of self-renewal may be due to *LAMA5* edits. We manually separated the clones into three different categories: low, intermediate, and high growth (Figures 3E, S3A, and S3B). Comparison of cell number per μFeature as well as doubling times between the high- and low-growth population revealed a significant difference between the populations (Student's two-tailed t test, *p* < 5 × 10^−5^) despite large variations in the slow-growth population (Figures S3B and S3C). The exact μFeature on ArrayEdit corresponding to the growth profile was identified using HCA, and we were able to easily pick clones of interest for expansion and further analysis. Twelve clones were isolated and expanded from each of the high- and intermediate-growth populations, and subsequently subjected to Sanger sequencing. As expected, all the high-growth clones maintained a wild-type genotype at all three sgRNA cut sites (Figures S3D and S3E, data not shown). All the intermediate-growth clones also sequenced correctly at all three loci, and expanded in a manner similar to high-growth populations after isolation, suggesting that there is some modest post-transfection transient variability in the growth rate of clones (Figures S3A and S3E). Therefore, we proceeded to focus our analysis on clones from the low-growth population.

We isolated, expanded, and genotyped low-growth clones identified by HCA on ArrayEdit for further characterization. The deletions generated by the sgRNA pool within hESC lines can easily be resolved on a standard agarose gel (Figure 4B). Agarose gels are intended as a semi-quantitative measure that can quickly screen clones to identify alleles that have undergone large-scale deletions. Due to differential repair pathways in edited cells such as microhomology-mediated end-joining (Bae et al., 2014), this quick assay may contain variable band sizes, and products require follow-on sequencing to confirm that the exact modification has occurred. Regardless, this gel assay was used to reveal cells that have been cut by two sgRNAs, producing either single or biallelic deletions. Agarose gels of isolated clones revealed 82% of clones had at least one large-scale deletion in the *LAMA5* allele (Figures 4C, 4D, and S3E). Of all selected clones, 46% contained a single allele edit, and 36% contained a biallelic edit, although not necessarily homozygous. The remaining 18% harbored wild-type genotypes, as determined by agarose gel screening assay. For the three sgRNAs transfected, three different large-scale deletions are predicted, all of which were observed (Figures S4A and S4B) suggesting that all sgRNAs within ArrayEdit were effective in cleaving genomic targets at similar rates. These results demonstrate significant improvement over prior methods used to obtain *LAMA5* gene-edited hESCs, where sequencing over 50 clones isolated via standard FACS methods yielded only one single allelic mutant and no biallelic modifications (Laperle et al., 2015) (Figure 4D).

On ArrayEdit, we selected five clones that exhibited a biallelic mutation pattern on the agarose screening gel for further analysis via sequencing. Of these five lines, three clones were found to contain near-precise, homozygous deletions between two sgRNAs (Figure 4E). The remaining two clones contained complex deletions around the sgRNA targets (Figure 4F). These lines were further subjected to off-target analysis as they are the most likely to have contained functional Cas9-sgRNA complexes and are therefore most likely to have experienced off-target activity. Off-target analysis on all five selected cell lines was performed by Sanger sequencing at three loci predicted by bioinformatics analysis (Hsu et al., 2013) to be the most likely off-target sites. This methodology does not preclude the possibility that there are other, unpredicted, gene edits. However, whole-genome sequencing would be required to find these modifications and was not pursued in this work. Sequencing at the most likely off-target loci revealed perfect alignment with the reference genotype in all five isolated biallelic clones, indicating that ArrayEdit can be used to generate targeted edits with minimal off-target effects (Figures 4G and S4C).

### Phenotypic Characterization of *LAMA5*-Edited hESCs

The five selected biallelic *LAMA5* gene-edited hESC lines were subsequently cultured on commonly used culture substrates: matrigel and laminin-111, both of which supply a low level of exogenous α-5 laminin but are insufficient to rescue the complete growth phenotype (Laperle et al., 2015). Importantly, all of the isolated clones expressed high levels of pluripotency markers, indicating that use of ArrayEdit does not lead to differentiation (Figure S5).

**Figure 5:**
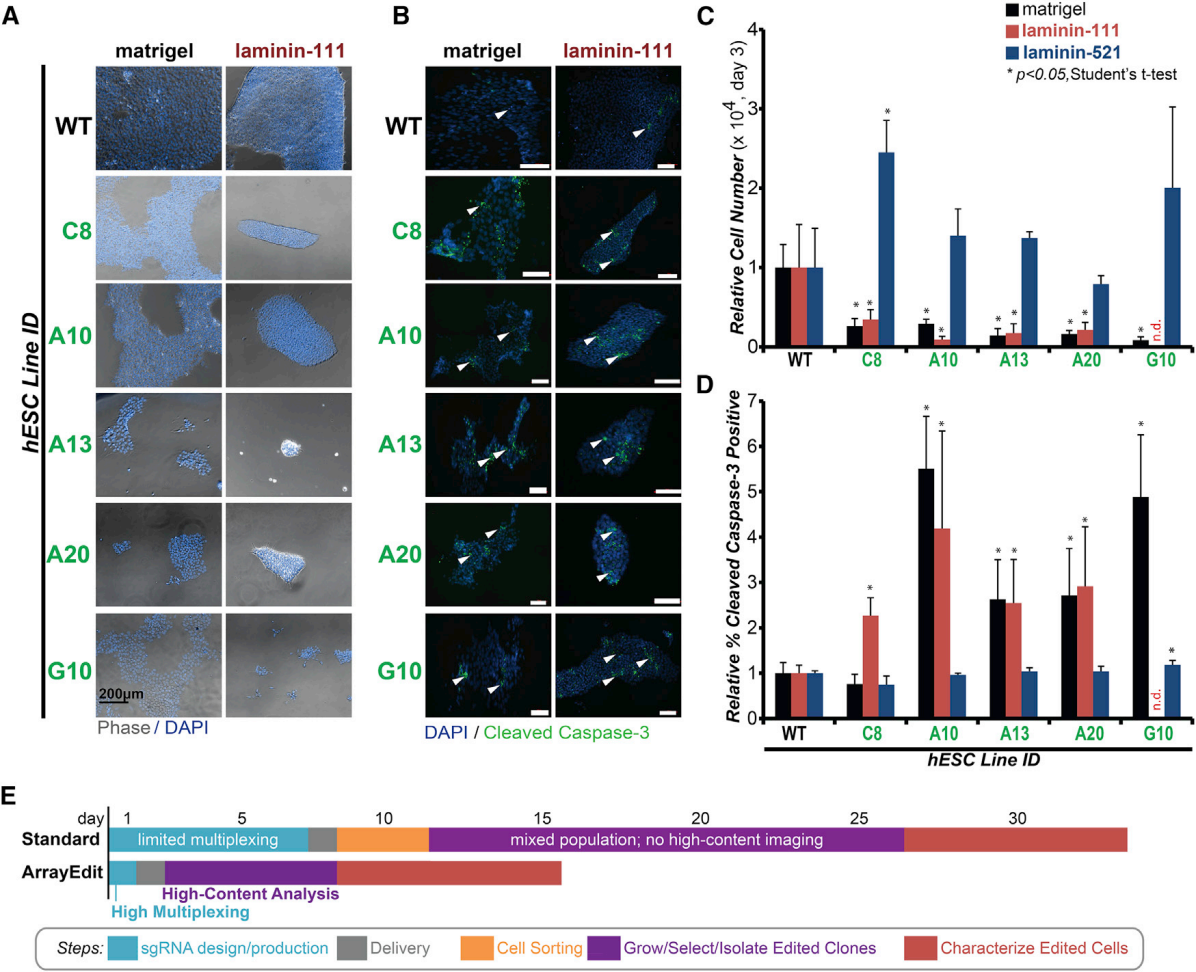
ArrayEdit Rapidly Produces Living, Well-Characterized, and Functional *LAMA5* Gene-Edited hESC Lines (A) Representative images of hESC colony formation of each biallelic edited line on both matrigel- and laminin111-coated substrates. Laminin-111 contains less matrix proteins thereby accentuating self-renewal defects generated from loss of α-5 laminin protein domains in the biallelic edited cells. Clone G10 notably displayed a drastic decrease in cell number on laminin-111. WT, wild-type. (B) Detection of apoptosis levels by immunocytochemistry of cleaved caspase-3. Each biallelic clone had detectable apoptotic cells (some are denoted by arrowheads). All scale bars represent 100 μm. (C and D) Mean number of cells (C) and cleaved caspase-3 positive cells (D) ±1 SD for each hESC clone on matrigel, laminin-111, laminin-521 coated substrates after 4 days in culture on various substrates as determined by flow cytometry (n = 4; independent experiments). On matrigel and laminin-111 substrates, each biallelic edited line was less dense than wild-type cells (Student's two-tailed t test, *p* < 0.05), indicating a functional defect in self-renewal. On laminin-521 substrates, each clone was at least as dense as wild-type cells, and had a similar number of apoptotic cells as wild-type except for G10. This may be due to complex deletions in the G10 clone. Clone G10 could not be detected (n.d.) due to a low cell number on laminin-111. Clone C8 had an in-frame mutation that may be partially rescued on the matrigel substrate and had a significantly higher number of cells on laminin-521. (E) Comparison of ArrayEdit against standard methods to produce gene-edited cells. ArrayEdit is approximately 2× faster and produces significantly more gene-edited cell lines (see Figure 4D). ArrayEdit reduces or eliminates several steps in the gene-editing workflow and additionally has important multiplexing and HCA capabilities. See also Figure S5.

After 3 days of culture, all five biallelic edited clones had significantly less cell numbers than the wild-type cells (n = 4; Student's two-tailed t test, *p* < 0.05) (Figures 5A and 5C) on both matrigel and laminin-111 substrates. All five clones had increased levels of apoptosis on laminin-111 compared with wild-type cells, while four of the five clones had higher levels of apoptosis on matrigel compared with wild-type (Figures 5B and 5D). Comparison of specific mutation to cell growth rate revealed that the clone with the earliest predicted stop codon in α-5 laminin, G10, with complex frameshift edits from exon 66 onward, had the least number of cells (Figure 5C) and among the highest levels of apoptosis (Figure 5D), while the clone with the latest predicted stop codon in α-5 laminin, C8, with only an in-frame deletion between exons 67 and 68, had levels of apoptosis similar to wild-type levels on matrigel (Figure 5D). The longer α-5 laminin produced by clone C8 may come together with an important component of matrigel to rescue apoptotic cells.

We then cultured all five *LAMA5* gene-edited lines on laminin-521, which has been proven to rescue growth phenotype in *LAMA5* knockouts (Laperle et al., 2015), to show that growth phenotype differences are a direct cause of the biallelic knockout. After 3 days in culture, four of the five lines had similar cell numbers to the wild-type, while one, C8, had a larger cell number (Figure 5C). Four of five clones also had levels of apoptosis, measured by cleaved caspase-3 percentage, similar to the wild-type cells. Clone G10, with its complex frameshift and early stop codon, had a slightly elevated percentage of apoptotic cells (Figure 5D).

Overall, these results indicate that *LAMA5* gene-edited cells on ArrayEdit exhibit the expected phenotype (Laperle et al., 2015), involving decreased rates of proliferation, high rates of apoptosis (Figures 5C and 5D), or a combination of both, on both matrigel and laminin-111. This phenotype was fully rescued by supplying a source of exogenous laminin-521, supporting the conclusion that precise edits were the only cause of the phenotype. ArrayEdit for *LAMA5* took approximately 15 days from conception and design to clonal expansion, which is substantially faster than the state-of-the-art methods that can take upward of a month (Figure 5E).

## Discussion

Here we tested whether a combination of improvements to the gene-editing workflow for hESCs would increase the efficiency and throughput of the overall process. One-pot transcription combined with μCP plates and HCA increased the multiplexing of edits within a single well or multiwell plate (three sgRNAs with three sgRNA combinations), increased the efficiency of isolating edited clones to 82%, and reduced the time necessary for a complete gene-editing workflow (15 days versus >30 days). To our knowledge, no other platform has combined these capabilities to gene edit cell lines. This combination of capabilities is most powerful when edited cells have phenotypes that can be distinguished with HCA. Lists of genes and sgRNA targets are now being assembled across pooled growth selection screens (Xu et al., 2015), and customized cells edited at many of these targets could be readily generated on ArrayEdit.

ArrayEdit is capable of identifying potentially edited cells by HCA in a non-destructive manner without single-cell dissociation or added transgenes, in contrast to FACS and sequencing-based methods to identify edited cells. This capability permits longitudinal temporal tracking of phenotypes within cells, with subcellular resolution, and avoids the need for subcloning, which typically adds at least 3–7 days to the workflow. ArrayEdit thus significantly reduces the culture and passaging required to isolate edited cells, enabling gene editing in human cell types that undergo substantial phenotypic changes with prolonged culture or primary cells that can only be cultured for few passages prior to senescence. In addition, ArrayEdit allows for the continuous culture of mutants that experience a deficit in self-renewal due to gene edits and would be out competed in standard culture. We found that 36% of our edited clones harbored biallelic editing of *LAMA5*, which is comparable with other CRISPR-Cas9 studies in human pluripotent stem cells (D’Astolfo et al., 2015, Ding et al., 2013, González et al., 2014, Mali et al., 2013). Efficiencies of editing on ArrayEdit would likely increase with advances in delivery modalities of DNA, RNA, and protein (Chiappini et al., 2015, D’Astolfo et al., 2015, Zuris et al., 2015) and application of additional selection pressure, such as drug selection (e.g., D’Astolfo et al., 2015, Merkle et al., 2015). The 82% efficiency that we demonstrate on ArrayEdit is below the 95%–100% purity of isolating edited cell lines by FACS frequently followed by drug selection, but our platform facilitates the identification of phenotypes by HCA, phenotypes that are very difficult or impossible to track with FACS. Furthermore, sorting can cause cellular stress, increasing cell death, which is especially challenging when isolating edited cells with a growth defect.

While we only implemented ArrayEdit for fluorescence loss and growth rate differences, the method employing HCA is extendable in principle to any image-based phenotype. Phenotypes could be defined by changes in uptake of cytoxicity dyes, live immunocytochemistry for cell surface or extracellular matrix markers, calcium flux dyes, or mitochondrial functional dyes (Taylor and Haskins, 2007). A distinguishable phenotype in edited cells may not be readily apparent by HCA in the pluripotent stem cell state. Hence, differentiation on ArrayEdit may be required to distinguish edited phenotypes. These capabilities on ArrayEdit seem possible, as arrayed neural organoid culture has already been achieved on μCP plates (Knight et al., 2015). More sophisticated computational methods could be easily implemented in our analysis pipeline in CellProfiler to prospectively identify imaging phenotypes that connect to proper or abnormal biological and epigenomic characteristics of edited cells (Singh et al., 2014). Because all edited clones share the same culture medium, the intra-well benchmarking of phenotypes on ArrayEdit could enable identification of phenotypes that may be lost due to noise or fluctuations in medium composition among culture wells or plates. Variations in signaling factors in the medium could also lead to hESC clones that become more or less lineage committed (Nazareth et al., 2013), opening a window to study the biological variability within clones that is difficult to ascertain during standard genotyping of clones. The stringent definition of phenotypes enabled by HCA with ArrayEdit will likely permit a more thorough characterization of the biological and functional consequences of various gene-editing protocols.

Current limitations of ArrayEdit arise from setting up the platform and screening for phenotypes in pluripotent cells. Although μCP is a straightforward technique, ArrayEdit is not a turn-key ready platform for many traditional biology or industrial laboratories who may need access to laser cutting or automated microscopy units. However, many commercial HCA instruments are available on the market, and our HCA pipeline can be readily employed using standard cloud-based computing or even a personal computer. The simple, versatile, and well-characterized μCP chemistry requires only standard laboratory equipment. The chemistry could also be flexibly modified to create various hydrophilic and hydrophobic areas on a single surface (McNulty et al., 2014, Sha et al., 2013), even well-of-the-well, water-in-oil culture platforms that are routinely used in pre-implantation embryo culture. Such chemically defined surfaces may be particularly attractive for clinical application in future work.

One-pot transcription of sgRNAs from PCR amplicons generated by oligonucleotide DNA primers produced clean and functional sgRNAs. One-pot transcribed sgRNAs can be rapidly designed and made from commercial vendors overnight with costs scaling with 20–60 base pair synthesis; the costs are anticipated to decrease over time (currently <$1 USD per sgRNA per experiment; see Table S4). In contrast to other methods that require the purchase of multiple oligonucleotides, our method requires only one unique oligonucleotide that can be synthesized in a multiwell plate format by commercial vendors, decreasing the setup time and the possibility of pipetting error. Errors in long (>60 nt), chemically synthesized oligonucleotides (up to 10%) have been observed (Liang et al., 2015), and our method notably avoids the use of long oligonucleotides by using a sequence-verified, synthesized, double-stranded DNA for the long universal region of the sgRNA. Our modular design also permits facile incorporation of additional RNA elements and devices (Nissim et al., 2014, Shechner et al., 2015). Furthermore, our method performed better than several previously described methods (González et al., 2014, Mali et al., 2013) (Figures 1D and E) and generated sgRNAs in less than 2 days. When analyzed via deep sequencing, one-pot sgRNAs had an efficiency of editing between 20% and 92% of mRNA transcripts after EB differentiation. Interestingly, many of the transcripts analyzed (5 of 7) had a higher percentage of in-frame mutations than would be caused by random chance (33%) (Shi et al., 2015). This may suggest that there were selection pressures in the EB cultures that modify the mutation spectrum observed.

We observed no off-target mutations in our edited cell lines at eight bioinformatically predicted sites by using one-pot sgRNAs, although we cannot rule out the presence of mutations at other loci. Because optimized Cas9 mRNA and protein delivery can reduce off-target mutagenesis (Kim et al., 2014), one-pot transcribed sgRNAs could be combined with these methods of delivering Cas9 to reduce the risk of off-target mutagenesis.

Overall, ArrayEdit provides a window into the editing process that could be useful in refining the specificity and versatility of CRISPR-Cas9 gene-editing techniques. In situ HCA could incorporate new optical and imaging techniques that monitor and perturb CRISPR-Cas9 editing. Inducible (Davis et al., 2015, Zetsche et al., 2015) and optical control (Hemphill et al., 2015, Nihongaki et al., 2015) of Cas9 activity has been demonstrated, and various spatial and temporal perturbations could be tested in future work on ArrayEdit. New nucleic acid probes could also visualize, or paint, specific loci of cells (Chen et al., 2013, Ma et al., 2015) within complex cellular structures on ArrayEdit, permitting the specific editing and isolation of edited clones with enhanced precision. ArrayEdit is fully compatible with existing screening platforms, so that small-molecule or other biological screens could be tested to enhance the efficacy of any desired gene-editing protocol. Also, ArrayEdit could be easily adapted to isolate cells with appropriate phenotypes after application of engineered, nuclease-dead, Cas9 protein fusions, such as those designed to activate or repress gene transcription. Finally, ArrayEdit will be likely applicable to many other human cell types, providing an attractive route to generating gene-edited human cells for a variety of industrial and preclinical purposes.

## Experimental Procedures

All work with human embryonic stem cell lines was carried out in accordance with institutional, national, and international guidelines and approved by the Stem Cell Research Oversight Committee at the University of Wisconsin-Madison.

### One-Pot Transcription of sgRNAs

IVT sgRNAs were synthesized in parallel in a 96-well plate within 1 day. The first step employed PCR with two chemically synthesized primers and a 125-bp double-stranded DNA template (Integrated DNA Technologies; see Figure S1A). Forward primers were ordered in 96-well plate format to enable high-throughput synthesis. PCR was performed using Phusion High-Fidelity Polymerase (New England Biolabs) according to the manufacturer’s protocols and was placed in the thermocycler at 98°C for 30 s followed by 35 cycles of 98°C for 5 s, 52°C for 10 s, and 72°C for 15 s before a final extension period of 72°C for 10 min. A truncated T7 promoter was included in the forward primer, which allowed the transcription of sgRNAs via a 37°C overnight reaction with a MEGAshortscript kit (Life Technologies).

### High-Content Image Acquisition and Analysis

Automated microscopy was performed using a Nikon Eclipse TI epifluorescent microscope and NIS Elements Advanced Research (V4.30) software. The ND acquisition 6D module was used to establish a 20 × 20 grid pattern such that one 10× image was taken at each μFeature and combined in a single file. Nikon Perfect Focus was used to ensure that all images were in the same Z-plane and in focus. Each image was then corrected for illumination defects using CellProfiler (Carpenter et al., 2006) and the number of nuclei was determined (Figure S2). Analysis was performed in a massively parallel manner using the Center for Throughput Computing (UW-Madison) and results were written to a local MySQL database. MySQL Workbench 6.1 CE was used to retrieve the data and join tables from time points on the basis of well and position.

## Author Contributions

J.C.S. and K.S. conceived and supervised the whole project. J.C.S. performed the major part of the research and wrote the manuscript with input from all authors. A.M., B.S., L.K., M.G., M.L., and R.P. provided technical support and comments on the manuscript.

## Accession Numbers

Raw reads from sequencing are available at NCBI Bioproject: PRJNA304252 and upon request.

## Supplemental Information

Document S1. Figures S1–S5, Tables S1, S2 and S4, Supplemental Experimental Procedures, and Supplemental Text

Table S3. μFeature Proliferation Data Ascertained by High Content Analysis over the Length of the Gene-Editing Experiment, Related To Figure 3

Document S2. Article plus Supplemental Information

## References

[bib1] Bae S., Kweon J., Kim H.S., Kim J.-S. (2014). Microhomology-based choice of Cas9 nuclease target sites. Nat. Methods.

[bib2] Baltimore D., Berg P., Botchan M., Carroll D., Charo R.A., Church G., Corn J.E., Daley G.Q., Doudna J.A., Fenner M. (2015). A prudent path forward for genomic engineering and germline gene modification. Science.

[bib3] Bosley K.S., Botchan M., Bredenoord A.L., Carroll D., Charo R.A., Charpentier E., Cohen R., Corn J., Doudna J., Feng G. (2015). CRISPR germline engineering—the community speaks. Nat. Biotechnol..

[bib4] Byrne S.M., Ortiz L., Mali P., Aach J., Church G.M. (2015). Multi-kilobase homozygous targeted gene replacement in human induced pluripotent stem cells. Nucleic Acids Res..

[bib5] Carpenter A.E., Jones T.R., Lamprecht M.R., Clarke C., Kang I.H., Friman O., Guertin D.A., Chang J.H., Lindquist R.A., Moffat J. (2006). CellProfiler: image analysis software for identifying and quantifying cell phenotypes. Genome Biol..

[bib6] Chen B., Gilbert L.A., Cimini B.A., Schnitzbauer J., Zhang W., Li G.-W., Park J., Blackburn E.H., Weissman J.S., Qi L.S., Huang B. (2013). Dynamic imaging of genomic loci in living human cells by an optimized CRISPR/Cas system. Cell.

[bib7] Chiappini C., De Rosa E., Martinez J.O., Liu X., Steele J., Stevens M.M., Tasciotti E. (2015). Biodegradable silicon nanoneedles delivering nucleic acids intracellularly induce localized in vivo neovascularization. Nat. Mater..

[bib8] D’Astolfo D.S., Pagliero R.J., Pras A., Karthaus W.R., Clevers H., Prasad V., Lebbink R.J., Rehmann H., Geijsen N. (2015). Efficient intracellular delivery of native proteins. Cell.

[bib9] Davis K.M., Pattanayak V., Thompson D.B., Zuris J.A., Liu D.R. (2015). Small molecule-triggered Cas9 protein with improved genome-editing specificity. Nat. Chem. Biol..

[bib10] Ding Q., Regan S.N., Xia Y., Oostrom L.A., Cowan C.A., Musunuru K. (2013). Enhanced efficiency of human pluripotent stem cell genome editing through replacing TALENs with CRISPRs. Cell Stem Cell.

[bib11] Doudna J.A. (2015). Genomic engineering and the future of medicine. JAMA.

[bib12] González F., Zhu Z., Shi Z.-D., Lelli K., Verma N., Li Q.V., Huangfu D. (2014). An iCRISPR platform for rapid, multiplexable, and inducible genome editing in human pluripotent stem cells. Cell Stem Cell.

[bib13] Harkness T., McNulty J.D., Prestil R., Seymour S.K., Klann T., Murrell M., Ashton R.S., Saha K. (2015). High-content imaging with micropatterned multiwell plates reveals influence of cell geometry and cytoskeleton on chromatin dynamics. Biotechnol. J..

[bib14] Hemphill J., Borchardt E.K., Brown K., Asokan A., Deiters A. (2015). Optical control of CRISPR/Cas9 gene editing. J. Am. Chem. Soc..

[bib15] Hsu P.D., Scott D.A., Weinstein J.A., Ran F.A., Konermann S., Agarwala V., Li Y., Fine E.J., Wu X., Shalem O. (2013). DNA targeting specificity of RNA-guided Cas9 nucleases. Nat. Biotechnol..

[bib16] Hsu P.D., Lander E.S., Zhang F. (2014). Development and applications of CRISPR-Cas9 for genome engineering. Cell.

[bib17] Kasap C., Elemento O., Kapoor T.M. (2014). DrugTargetSeqR: a genomics- and CRISPR-Cas9–based method to analyze drug targets. Nat. Chem. Biol..

[bib18] Kim S., Kim D., Cho S.W., Kim J., Kim J.-S. (2014). Highly efficient RNA-guided genome editing in human cells via delivery of purified Cas9 ribonucleoproteins. Genome Res..

[bib19] Knight G.T., Sha J., Ashton R.S. (2015). Micropatterned, clickable culture substrates enable in situ spatiotemporal control of human PSC-derived neural tissue morphology. Chem. Commun. (Camb)..

[bib20] Laperle A., Hsiao C., Lampe M., Mortier J., Saha K., Palecek S.P., Masters K.S. (2015). α-5 laminin synthesized by human pluripotent stem cells promotes self-renewal. Stem Cell Rep..

[bib21] Liang X., Potter J., Kumar S., Zou Y., Quintanilla R., Sridharan M., Carte J., Chen W., Roark N., Ranganathan S. (2015). Rapid and highly efficient mammalian cell engineering via Cas9 protein transfection. J. Biotechnol..

[bib22] Lin S., Staahl B.T., Alla R.K., Doudna J.A. (2014). Enhanced homology-directed human genome engineering by controlled timing of CRISPR/Cas9 delivery. eLife.

[bib23] Ma H., Naseri A., Reyes-Gutierrez P., Wolfe S.A., Zhang S., Pederson T. (2015). Multicolor CRISPR labeling of chromosomal loci in human cells. Proc. Natl. Acad. Sci. USA.

[bib24] Mali P., Yang L., Esvelt K.M., Aach J., Guell M., DiCarlo J.E., Norville J.E., Church G.M. (2013). RNA-guided human genome engineering via Cas9. Science.

[bib25] McNulty J.D., Klann T., Sha J., Salick M., Knight G.T., Turng L.-S., Ashton R.S. (2014). High-precision robotic microcontact printing (R-μCP) utilizing a vision guided selectively compliant articulated robotic arm. Lab. Chip.

[bib26] Merkle F.T., Neuhausser W.M., Santos D., Valen E., Gagnon J.A., Maas K., Sandoe J., Schier A.F., Eggan K. (2015). Efficient CRISPR-Cas9-mediated generation of knockin human pluripotent stem cells lacking undesired mutations at the targeted locus. Cell Rep..

[bib27] Miyaoka Y., Chan A.H., Judge L.M., Yoo J., Huang M., Nguyen T.D., Lizarraga P.P., So P.-L., Conklin B.R. (2014). Isolation of single-base genome-edited human iPS cells without antibiotic selection. Nat. Methods.

[bib28] Nazareth E.J.P., Ostblom J.E.E., Lücker P.B., Shukla S., Alvarez M.M., Oh S.K.W., Yin T., Zandstra P.W. (2013). High-throughput fingerprinting of human pluripotent stem cell fate responses and lineage bias. Nat. Methods.

[bib29] Nihongaki Y., Kawano F., Nakajima T., Sato M. (2015). Photoactivatable CRISPR-Cas9 for optogenetic genome editing. Nat. Biotechnol..

[bib30] Nissim L., Perli S.D., Fridkin A., Perez-Pinera P., Lu T.K. (2014). Multiplexed and programmable regulation of gene networks with an integrated RNA and CRISPR/Cas toolkit in human cells. Mol. Cell.

[bib31] Sanjana N.E., Shalem O., Zhang F. (2014). Improved vectors and genome-wide libraries for CRISPR screening. Nat. Methods.

[bib32] Schumann K., Lin S., Boyer E., Simeonov D.R., Subramaniam M., Gate R.E., Haliburton G.E., Ye C.J., Bluestone J.A., Doudna J.A., Marson A. (2015). Generation of knock-in primary human T cells using Cas9 ribonucleoproteins. Proc. Natl. Acad. Sci. USA.

[bib33] Sha J., Lippmann E.S., McNulty J., Ma Y., Ashton R.S. (2013). Sequential nucleophilic substitutions permit orthogonal click functionalization of multicomponent PEG brushes. Biomacromolecules.

[bib34] Shalem O., Sanjana N.E., Hartenian E., Shi X., Scott D.A., Mikkelsen T.S., Heckl D., Ebert B.L., Root D.E., Doench J.G., Zhang F. (2014). Genome-scale CRISPR-Cas9 knockout screening in human cells. Science.

[bib35] Shechner D.M., Hacisuleyman E., Younger S.T., Rinn J.L. (2015). Multiplexable, locus-specific targeting of long RNAs with CRISPR-Display. Nat. Methods.

[bib36] Shi J., Wang E., Milazzo J.P., Wang Z., Kinney J.B., Vakoc C.R. (2015). Discovery of cancer drug targets by CRISPR-Cas9 screening of protein domains. Nat. Biotechnol..

[bib37] Singh S., Carpenter A.E., Genovesio A. (2014). Increasing the content of high-content screening an overview. J. Biomol. Screen.

[bib38] Smurnyy Y., Cai M., Wu H., McWhinnie E., Tallarico J.A., Yang Y., Feng Y. (2014). DNA sequencing and CRISPR-Cas9 gene editing for target validation in mammalian cells. Nat. Chem. Biol..

[bib39] Sternberg S.H., Doudna J.A. (2015). Expanding the biologist’s toolkit with CRISPR-Cas9. Mol. Cell.

[bib40] Taylor D.L., Haskins J.R. (2007). High Content Screening: A Powerful Approach to Systems Cell Biology and Drug Discovery.

[bib41] Wang T., Wei J.J., Sabatini D.M., Lander E.S. (2014). Genetic screens in human cells using the CRISPR-Cas9 system. Science.

[bib42] Xu H., Xiao T., Chen C.-H., Li W., Meyer C., Wu Q., Wu D., Cong L., Zhang F., Liu J.S. (2015). Sequence determinants of improved CRISPR sgRNA design. Genome Res..

[bib43] Yang L., Guell M., Byrne S., Yang J.L., Angeles A.D.L., Mali P., Aach J., Kim-Kiselak C., Briggs A.W., Rios X. (2013). Optimization of scarless human stem cell genome editing. Nucleic Acids Res..

[bib44] Zetsche B., Volz S.E., Zhang F. (2015). A split-Cas9 architecture for inducible genome editing and transcription modulation. Nat. Biotechnol..

[bib45] Zuris J.A., Thompson D.B., Shu Y., Guilinger J.P., Bessen J.L., Hu J.H., Maeder M.L., Joung J.K., Chen Z.-Y., Liu D.R. (2015). Cationic lipid-mediated delivery of proteins enables efficient protein-based genome editing in vitro and in vivo. Nat. Biotechnol..

