# Supplementary material for: High-Content Analysis of CRISPR-Cas9 Gene-Edited Human Embryonic Stem Cells
Source: Stem Cell Reports. 2016 Jan 12;6(1):109–20. doi: 10.1016/j.stemcr.2015.11.014 (PMC4720027; doi:10.1016/j.stemcr.2015.11.014)
Supplement: Document S1. Figures S1–S5, Tables S1, S2 and S4, Supplemental Experimental Procedures, and Supplemental Text [file mmc1.pdf]

Stem Cell Reports, Volume *6*

Supplemental Information

High-Content Analysis of CRISPR-Cas9 Gene-Edited Human Embryonic Stem Cells

Jared Carlson-Stevermer, Madelyn Goedland, Benjamin Steyer, Arezoo Movaghar, Meng Lou, Lucille Kohlenberg, Ryan Prestil, and Krishanu Saha

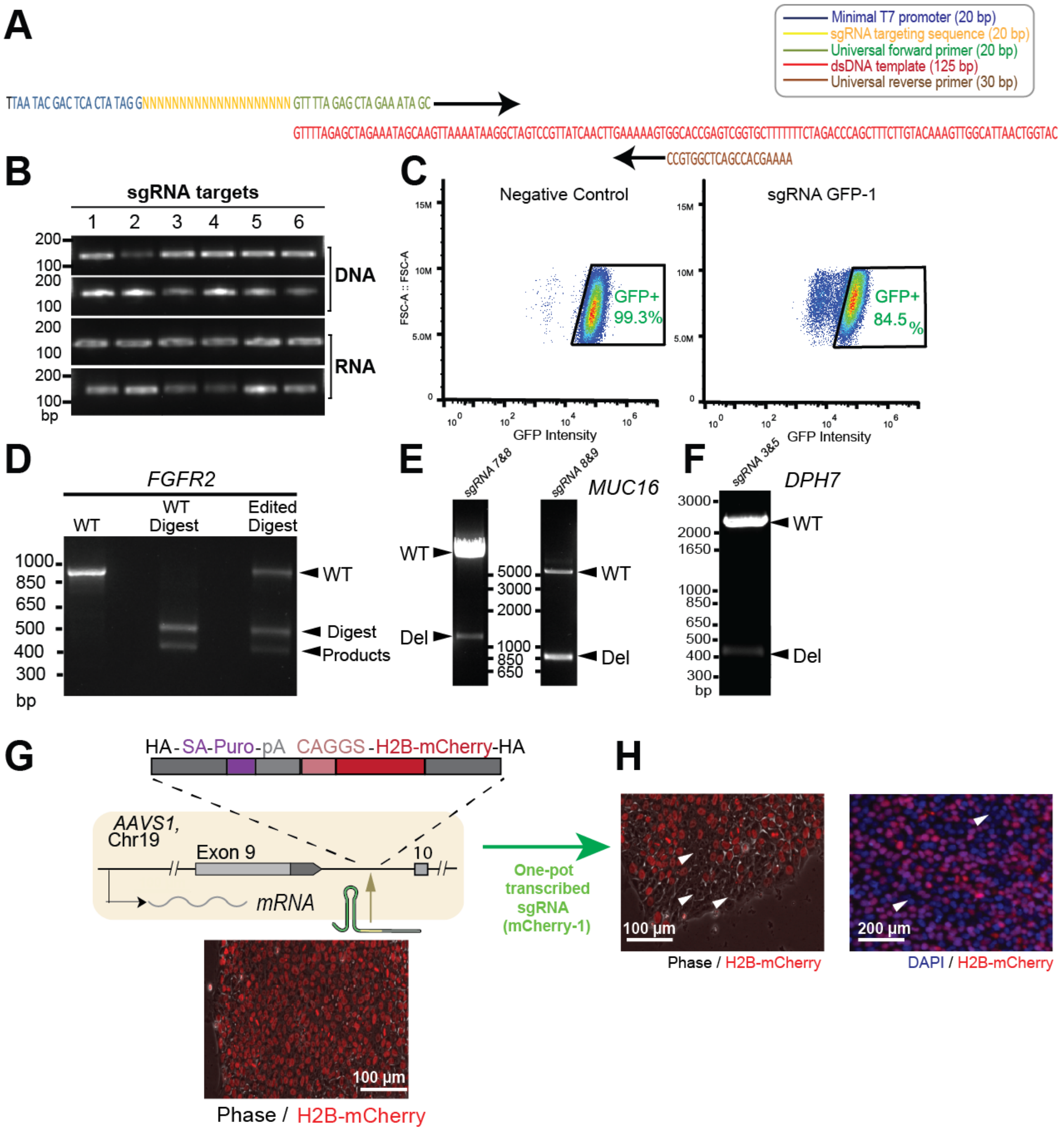

Figure S1. Synthesis and functionality of "one-pot" transcribed single guide RNAs, Related to Figure 1.

(A) Schematic of the PCR step for "one-pot" transcription of sgRNA. The forward primer defines the genomic target (yellow) of editing by Cas9, while the reverse primer encodes the constant regions of the sgRNA. Truncated T7 promoter (blue) is used for in vitro transcription. **(B)** Gels with each column showing DNA and RNA products from one-pot reactions performed twice with six distinct primer sets (targets 1-3: *MCHERRY-1-3*; 4-6: *GFP-1-3*). Each reaction occurred in one well of a 96-well plate. *Top two rows:* Agarose gel of DNA generated by PCR using primers and template shown in part (A). Products are of consistent length and yield, regardless of target. *Bottom two rows:* Denaturing agarose gel following one-pot transcription of DNA shown in top. Products are of a consistent length and yield. **(C)** Flow cytometry results knocking out a constitutive GFP reporter in human embryonic kidney (HEK) cells using sgRNA GFP-1 (>10,000 events; replicate experiments). **(D)** Restriction fragment length polymorphism assay of gene editing at the *FGFR2* locus. Gene modification removes an endogenous PvuI site in *FGFR2*. WT denotes wildtype sequence. WT digest: WT PCR digested using PvuI. WT band is completely removed in digested lane. Edited digest denotes hESCs transfected with sgRNA FGFR2-1. WT band remains suggesting the removal of the PvuI cut site via gene editing. **(E, F)** Large-scale genome edits in HEK cells in (E) *MUC16* and (F) *DPH7*. The presence of a WT band and a smaller second band show that 2-4 kb have been cut out of a population in the analyzed cells. **(G)** Generation of WA09-H2B-mCherry line. *Top*: Donor plasmid for histone2B-mCherry insertion used. Constitutive expression from a safe-harbour, intronic *AAVS1* locus was employed: CAAGS: synthetic promoter, Puro: puromycin resistance, SA: splice acceptor, HA: homology arm; pA: poly(A) tail. *Bottom*: Overlay of phase and red fluorescence channels of WA09 hESCs with transgene. All nuclei contain red fluorescence. **(H)** Images of WA09-H2B-mCherry hESCs on standard culture plates four days after electroporation with Cas9-encoding plasmid and one-pot sgRNAs against mCherry transgene. Arrowheads: cells that lost mCherry fluorescence due to gene-editing.

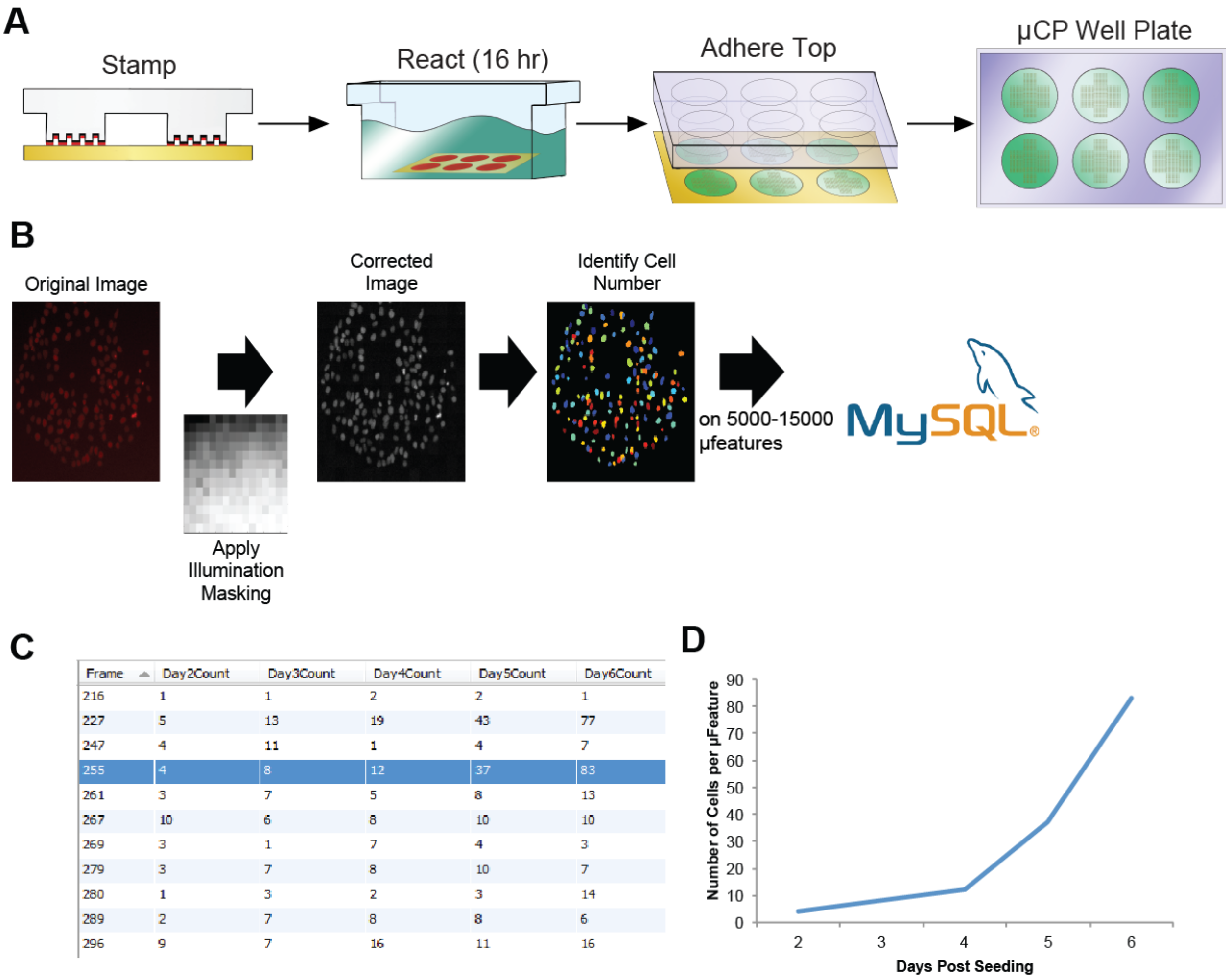

Frame	Day2Count	Day3Count	Day4Count	Day5Count	Day6Count
216	1	1	2	2	1
227	5	13	19	43	77
247	4	11	1	4	7
255	4	8	12	37	83
261	3	7	5	8	13
267	10	6	8	10	10
269	3	1	7	4	3
279	3	7	8	10	7
280	1	3	2	3	14
289	2	7	8	8	6
296	9	7	16	11	16

Figure S2. Microcontact printing permits isolation of gene-edited hESCs on ArrayEdit, Related to Figure 3.

(A) Schematic of µcontact printing methodology. Alkanethiol self-assembled monolayers are deposited onto gold-coated glass via a polydimethylsiloxane (PDMS) stamp. Stamped glass was then submerged in poly (ethylene-glycol) (PEG) solution overnight. Finally, reacted glass was attached to a standard well plate. **(B)** Detail of image analysis pipeline. First, an illumination mask is applied in CellProfiler to even out bright and dark areas. Second, the number of nuclei is identified. For each µFeature, that number, as well as spatial location, is exported to a MySQL database. **(C)** Representative table of growth data from MySQL Workbench. **(D)** Example of tracking growth data using highlighted data (blue in part c) from one µFeature over time.

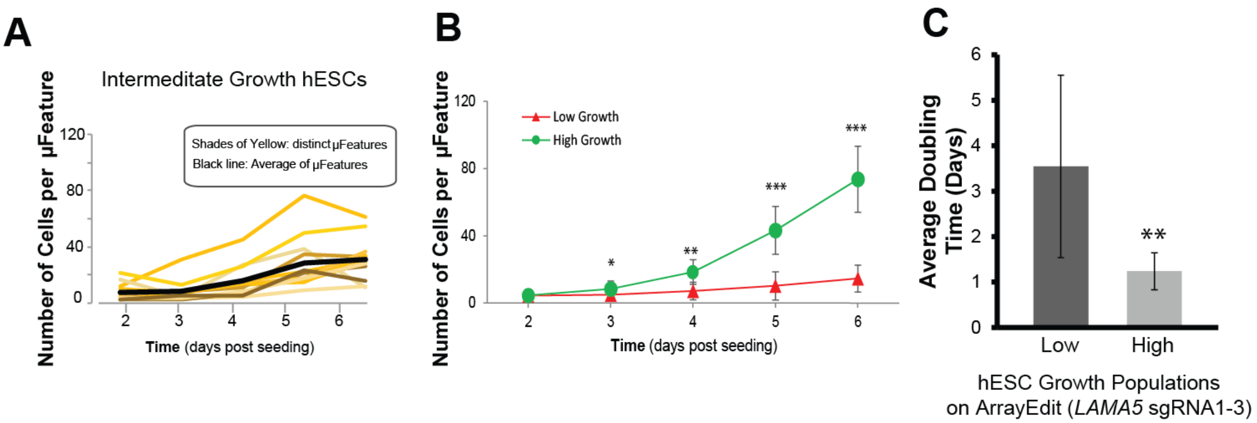

D

hESC Growth Population on ArrayEdit (*LAMA5* sgRNA1-3)	***LAMA5* around sgRNA1 target**	
	TTCAGCGTGTCCATCTCGATGCAGCCCCGGTAGCCGGGGAAGCGAAGCAGGGGAGGGGGCTGTGGG	**WT**
Intermediate Growth Clone 1	TTCAGCGTGTCCATCTCGATGCAGCCCCNGTAGCCGGGGAAGCGAAGCAGGGGAGGGGNCTGTGGG	
Intermediate Growth Clone 2	TTCAGCGTGTCCATCTCGATGCAGCCCCGGTAGCCGGGGAAGCGAAGCAGGGGAGGGGGCTGTGGG	
Intermediate Growth Clone 3	TTCAGCGTGTCCATCTCGATGCAGCCCCGGTAGCCGGGGAAGCGAAGCAGGGGAGGGGGCTGTGGG	
Intermediate Growth Clone 4	TTCAGCGTGTCCATCTCGATGCAGCCCCGGTAGCCGGGGAAGCGAAGCAGGGGAGGGGGCTGTGGG	
Intermediate Growth Clone 5	TTCAGCGTGTCCATCTCGATGCAGCCCCGGTAGCCGGGGAAGCGAAGCAGGGGAGGGGGCTGTGGG	
High Growth Clone 1	TTCAGCGTGTCCATCTCGATGCAGCCCCNGTAGCCGGGGAAGCGAAGCAGGGGAGGGGNCTGTGGG	
High Growth Clone 2	TTCAGCGTGTCCATCTCGATGCAGCCCCGGTAGCCGGGGAAGCGAAGCAGGGGAGGGGGCTGTGGG	
High Growth Clone 3	TTCAGCGTGTCCATCTCGATGCAGCCCCGGTAGCCGGGGAAGCGAAGCAGGGGAGGGGGCTGTGGG	
High Growth Clone 4	TTCAGCGTGTCCATCTCGATGCAGCCCCGGTAGCCGGGGAAGCGAAGCAGGGGAGGGGGCTGTGGG	
High Growth Clone 5	TTCAGCGTGTCCATCTCGATGCAGCCCCGGTAGCCGGGGAAGCGAAGCAGGGGAGGGGGCTGTGGG	

E

Population	Wildtype	Single Allelic Edit	Biallelic Edit
High	12/12 (100%)	0/12 (0%)	0/12 (0%)
Medium	12/12 (100%)	0/12 (0%)	0/12 (0%)
Low	4/22 (18%)	10/22 (46%)	8/22 (36%)

Figure S3. LAMA5-edited hESC lines grouped by high-content growth analysis on ArrayEdit, Related to Figure 4.

(A) Growth curves for intermediate growth rate grouped cells within 12 μFeatures on ArrayEdit over 5 consecutive days. See Fig. 3E for curves of the low and high growth populations. Yellow lines are individual populations. Black line is the average of all populations in the intermediate group. **(B)** Growth curves of the high and low growth populations. Data are represented as mean ± 95% C.I. from 12 independent μFeatures on ArrayEdit. Starting at day 3, the populations have significantly different number of cells (Student's t-test, *p<0.05, **$p<5x10^{-5}$, ***$p<5x10^{-10}$) **(C)** Doubling times for both high and low growth population observed on ArrayEdit. Data are represented as mean ± 95% C.I. from 12 independent μFeatures on ArrayEdit. Low growth populations have significantly higher doubling times than high growing populations ($p<5x10^{-5}$, Student's t-test). **(D)** Sanger sequencing analysis of representative intermediate and high growth hESC lines isolated from ArrayEdit. Wildtype is denoted on top, and the hESC lines are below. sgRNA targets are denoted in red and PAM in blue. No edits were observed in these cells, as well as all of the other 14 clones isolated from the intermediate and high growth populations. **(E)** Table of observed genotypes in the high, intermediate, and low growth groups. No edits were observed in either the high or intermediate growth groups.

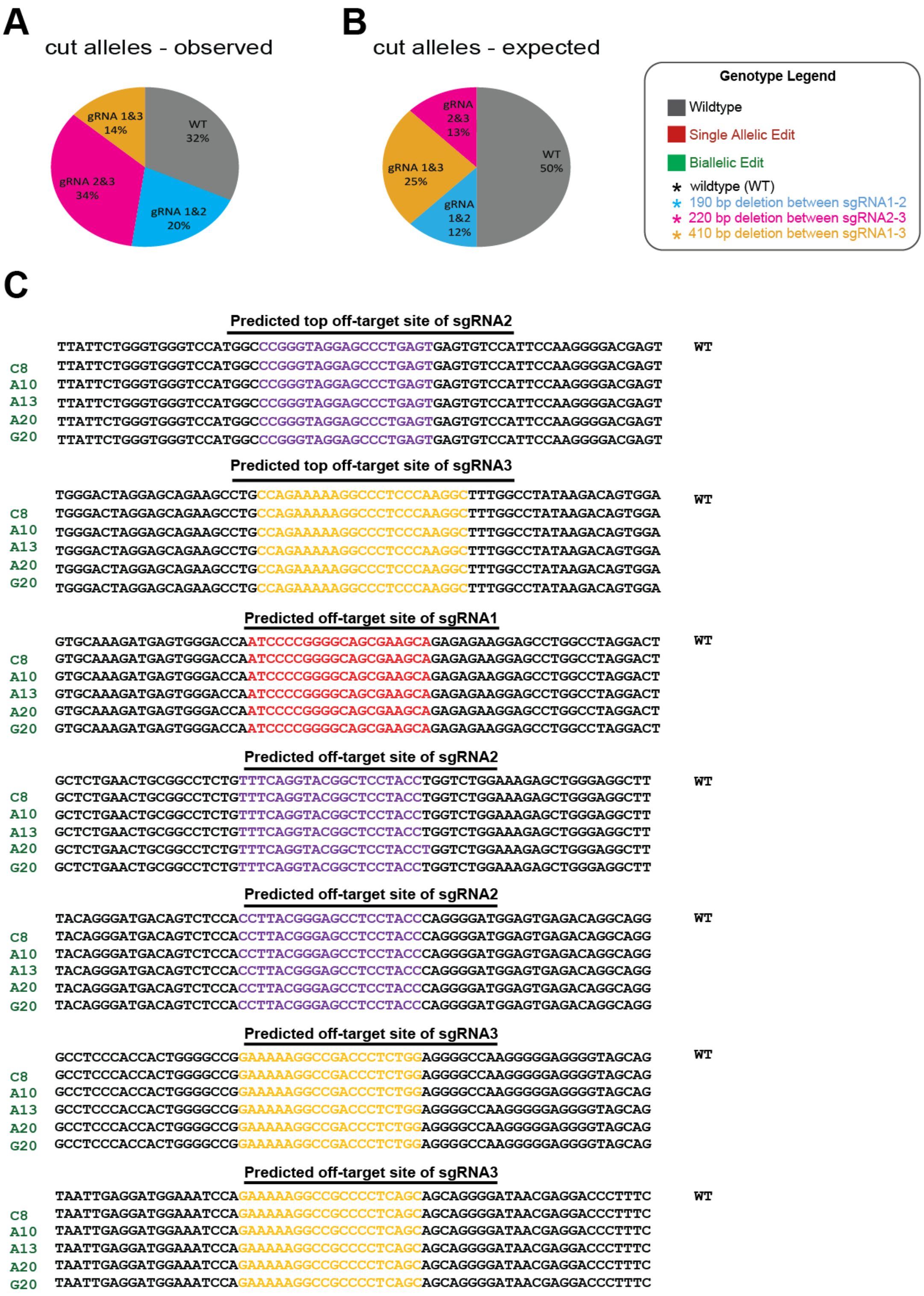

Figure S4. Genotypes observed in edited hESC lines isolated from ArrayEdit, Related to Figure 4.

(A) Summary of genotypes obtained from hESC lines isolated after gene-editing using ArrayEdit or standard procedures (from screening gels in Figures 4C; see text), to edit *LAMA5*. **(B)** Calculated distribution of cut alleles from a probability tree. Construction of the tree made the following assumptions: all sgRNAs cut at the same frequency and that any sgRNA that enters the cell will generate edits. **(C)** Sanger sequencing around loci of top predicted off-target sites of LAMA5 sgRNAs 1, 2 and 3 as determined by bioinformatics. Potential off-target sites are colored. All sequenced clones show perfect alignment to reference genotype at the off-target sites.

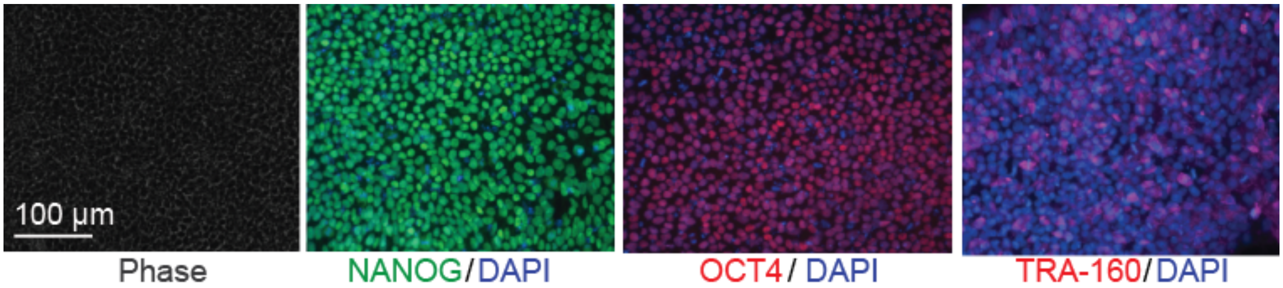

Figure S5. Pluripotency characterization of hESCs after isolation from ArrayEdit, Related to Figure 5. Immunocytochemical assay for pluripotency proteins (Oct4, Sox2, TRA-1-60) within hESCs after isolation from ArrayEdit. Live, gene-edited hESCs isolated from platform retain pluripotent marker expression over >10 passages.

SUPPLEMENTAL TABLES

Table S1. Primer Sequences Used In This Study, Related To Figure 1. All sequences are listed as 5' to 3'.

Reverse primer, RNAtracrR

AAAAGCACCGACTCGGTGCC

dsDNA Template

GTTTTAGAGCTAGAAATAGCAAGTTAAAATAAGGCTAGTCCGTTATCAACTTGAAAAAGTG
GCACCGAGTCGGTGCTTTTTTTCTAGACCCAGCTTTCTTGTACAAAGTTGGCATTAACTGGTAC

Genomic Amplification

**mCherry**	**LAMA5 Genomic**
AAGGGCGAGGAGGATAACATGG	CCTCTGCCCTCAGGACTCT
TTGTACAGCTCGTCCATGCCG	GGCCAGCTCCAGATCATTGT
**MUC16**	**LAMA5 Off-target 1**
AGTGAGGACACTCAGAACCAC	TCAGAGCAGTTGCCTTCCCA
ATGTTGGGGTCTCAGATGGTG	TTTGGCATCCTTCGGCCTT
**DPH7**	**LAMA5 Off-target 2**
ACTGCAGCCCCGTTGTTAAG	GAGTCTCGGCAGATGGTAGC
AAAGGTCTTCAAGATCCCAACA	TCAGCGTGAGCACACTCATT
**CDH5**	**LAMA5 Off-target 3**
GACTCCTCCAGCTTCACCA	CTCTACACCAAAGAGCGGCA
CACGGACGCATTGAACAACC	GCAATGCCAGATGGGTCAAC
**CDH20**	**LAMA5 Off-target 4**
ATCCAGACAGTGAGTGCGGT	ACACTGCATTCTGAAGCCCA
CCTCAGGAGCCAAGCTGTAG	GCTGGCCGGCTGAGG
**FOXP2**	**LAMA5 Off-target 5**
AGCATCTGCTCAGCCTTCAG	TCCGCAAGGGTTTCTTGGTA
TTGAGGCAGCGATTGGACAG	TTTCAGGAGCTGTTCTGGGAC
**NANOG**	**LAMA5 Off-target 6**
AAGGCCTCAGCACCTACCTA	CAGGCCAAATGTCCCATCCT
GAAGGTTCCCAGTCGGGTTC	ACAGTACCAGGCTTCGTGTG
**OCT4**	**LAMA5 Off-target 7**
GTGGTCCGAGTGTGGTT	ACATGGGCCATAGGAGATGC
GAAAGGAGACCCAGCAG	CCCAGGTGGGACTCTCAAAC
**PAX6**	**LAMA5 Off-target 8**
AAGCAAAATAGCCCAGTATAAG	TCCCTCCCTTTGATGGGTTG
TATGTTATCGTTGGTACAGACC	CCGCTGATTGAAAGCCTCTT
**POU4F1**	**LAMA5 Off-target 9**
CCTCACCCGCATATGCACAG	CCGCTACTTGCCAAACACAT
CCCGGACGGCATGTTCA	CTCCATGTCCAGGGTTCTGC

Table S2. Genomic targets for sgRNAs produced using one-pot transcription, Related To Figure 1. All sequences are listed as 5' to 3'. (Labels for each sgRNA target in text are in parentheses.) Synthesized forward primers were `TTAATACGACTCACTATAGG-Target_sequence_below-GTTTTAGAGCTAGAAATAGC`.

LAMA5
GTAGCCGGGGAAGCGAAGCA (LAMA5-1)
GCTCACGGACGGCTCCTACC (LAMA5-2)
GCAGTGGGACGGCCTTTTTC (LAMA5-3)

MCHERRY
GGAGCCGTACATGAACTGAG (mCherry-1)
CTCGAACTCGTGGCCGTTCA (mCherry-2)
GCGGTCTGGGTGCCCTCGTA
CGCCCTCGATCTCGAACTCG
GGGCGAGGGCCGCCCCTACG
AGTAGTCGGGGATGTCGGCG (mCherry-3)
GCGTTCGTACTGTTCCACGA
CTGTCCCCTCAGTTCATGTA

GFP
GACCAGGATGGGCACCACCC (GFP-1)
GCACGGGGCCGTCGCCGATG (GFP-2)
GTGAACCGCATCGAGCTGAA (GFP-3)
CTCGAACTCGTGGCCGTTCA

NANOG
GTCTTCAGGTTGCATGTTCA
AAGTCACTGGCAGGAGAATT
GTATTCGTATTGTTTGGGAT
GTGCTAATCTTTGTAGAAAG
CAGTCGGATGCTTCAAAGCA
CTTCAAAGCAAGGCAAGCTT
AGAGAAGAGTGTCGCAAAAA
GATGCCTGGTGAACCCGACT
CTTTACTCTTCCTACCACCA (NANOG-1)

SOX2
GTGCCGTTAATGGCCGTGCC
GTTCCGGCACCTCGGCGCCG
GTGCCCGGCACGGCCATTAA
ACAACTACGGAAAATAAAGG
CAACTACGGAAAATAAAGGG

OCT4
ATCGCTTGCCCTTCTGGCGC
TGCTTGATCGCTTGCCCTTC
GAACTTAATCCCAAAAACCC
CCTGTCCCCCATTCCTAGAA
TCTCTTTCGGGCCTGCACGA
CAGCGACTATGCACAACGAG (OCT4-1)

FGFR2
GGCCGGTGAGGCGATCGCTC
GTGGCCTCGGTACCGGAGTG (FGFR2-1)
CACCAGAGCGATCGCCTCAC

PAX6
TTATTATAGAAATCATTCTG
CGAGACAGATTACTGTCCGA (PAX6-1)

OTX2
GCCTAGCAGTAAAGAGACAT
ACCAATGTCTCTTTACTGCT

MUC16
GTCACCAACCGTGATACAGC
GCTATCGTCTTTGGTTCACC
GTGGGGACTTAGTCAGTGTT
GACCCCACTGGTCACTATTT
GAAAACAGATGGGGTTGTCC
GCCAATGAGGATGTCGTTTC
GGTTTTACTGAAACCCCTGA (MUC16-7)
TCACCCCACTGACAACCACG (MUC16-8)
CGGGAGCTCTGACATCCTGA (MUC16-9)

CDC42
AAGTGTGTTGTTGTGGGCGA
GGTGGAGAACCATATACTCT
TCTGTTTGTGGATAACTCAG
CACAACAAACAAATTTCCAT
GCAGTCACAGTTATGATTGG
ATTTCTAGTCTGTTTTTCAG

DPH7
GCACCACTCCACCGAGTCCG
TTCTTTGGACCTCGACCAGA
ATCCATACAACTGCTCCGCC (DPH7-3)
TTTCCCAGTGGACCAATCTA
ATGGCAGGCACATCAATTCG (DPH7-5)
GGCGACGATGGCCTTCTGAG
CCAGGATGTGCTCCCGATGA
ACGAAACATGAAGCAGCCGT
GGTCTCTTGCCTCGGTGCCT
GTTCACGCCACAGTCACGTG

CDH20
TTACCTTGCCGACATACAAA
AGAAATGCTGACCATTGCGT (CDH20-1)

CDH5
ACTGGCCTGTGTTCACGCAT

FMR1
CCTTCCTTCCAATAATTCAA

FOXP2
GGACTCATCTCCATTCCACC

GAPDH
TATTGGGCGCCTGGTCACCA (GAPDH-1)
ACTGTGGCGTGATGGCCGCG

POU4F1
GGGCCACCTGTCGCACCCCG

Table S3. μFeature proliferation data ascertained by high-content analysis over the length of gene-editing experiment, Related To Figure 3. Each row indicates a different μFeature within ArrayEdit in Figure 3D-E.

See Supplementary Spreadsheet (XLS file).

Table S4. Nucleic acid-related reagent cost estimates for implementing ArrayEdit, Related to Discussion. Standard protocol uses cloning of sgRNA into a plasmid backbone (e.g. Mali et al., *Science*, 2013).

	ArrayEdit	Standard
gBlock	$2.11	--
Forward Primer	$12.00	$12.00
Reverse Primer	--	$12.00
Polymerase	$0.84	$0.84
IVT	$2.12	--
Restriction Enzyme	--	$0.31
Gibson Assembly	--	$15.90
Competent Bacteria	--	$24.00
Miniprep	--	$1.63
Reagent Cost	$17.07	$66.68
Number of uses	20	2
Cost per experiment	**$0.85**	**$33.34**

SUPPLEMENTAL EXPERIMENTAL PROCEDURES

One-pot transcribed sgRNA design

For each desired gene edit, potential sgRNA targets were identified using the crispr.mit.edu design tool and selected to reduce the number of off-target effects as determined by bioinformatics (Hsu et al., 2013). In general, high quality selected sgRNA targets scored above 80 on the crispr.mit.edu design tool.

RNA quantification and characterization

For quantification and characterization of sgRNAs, purification was performed with the MEGAclear transcription kit (Life Technology) and quantified on a Nanodrop 2000. An Aligent 2100 Bioanalyzer was used according to manufacturer protocols to determine the uniformity of the produced sgRNA. Due to consistent yields post-purification, approximate molar quantities were used for transfections based off of sgRNA volume used.

Cell culture

WA09 hESCs (WiCell, Madison, WI) and HUES9-iCas9 (generously provided by the lab of Dr. Danwei Huangfu, Sloan-Kettering Institute, New York, NY) were maintained in E8 medium on Matrigel (WiCell) coated tissue culture polystyrene (TCPS) plates (BD Falcon) (Chen et al., 2011). Cells were passaged every 3-4 days at a 1:6 ratio using Versene solution (Life Technologies). For experimental procedures on μCP plates, cells were singularized using Accutase (StemPro) and seeded on to plates in mTESR1 medium (WiCell) containing 10 μM ROCK inhibitor (Y27632, Selleckchem).

Human embryonic kidney cells (293T) were maintained between passage 15-40 in Growth medium containing DMEM (Life Technologies), 10% v/v FBS (Life Technologies), 2mM L-Glutamine (Life Technologies), and 50 U/mL Penicillin-Streptomycin (Life Technologies). Cells were passaged 1:40 with Trypsin-EDTA (Life Technologies) onto Gelatin-A (Sigma) coated plates.

Cell culture substrate coatings

24-well tissue cultue polystyrene plates (TCPS) were coated with matrigel, laminin-111, or laminin-521. Matrigel (WiCell) diluted in DMEM-F12 (Life Technologies) and coated onto TCPS at a density of 8.7 $\mu g/cm^2$ overnight at 37ºC. Laminin-111 (Cultrex) was diluted in PBS and coated onto poly-ornithine-coated TCPS well at a density of 1 $\mu g/cm^2$ then incubated overnight at 37ºC. Laminin-521 (BioLamina) was diluted in 1x DBPS with Ca^{2+} and Mg^{2+} and coated onto poly-ornitihine-coated TCPS well at a density of 1.5 $\mu g/cm^2$ then incubated at 37ºC for 2 hours.

Nucleic acid delivery to human cell lines

Nucleic acids were electroporated into two hESC lines: one harboring a mCherry transgene, WA09-H2B-mCherry (**Fig. S1**) and another harboring an inducible Cas9 transgene, HUES9-iCas9. Doxycycline was added to inducible Cas9 line at 5 μM 48 hours prior to experiments and both lines were treated with 10 μM ROCK inhibitor (Y27632, Selleckchem) 24 hours prior to experiments. Electroporation was performed using a GenePulser Xcell, with attached CE module (BioRad). Cells were first singularized using Accutase (StemPro), then 10^6 cells were centrifuged and resuspended in 400 μL mTESR1 medium containing 5 μg of each sgRNA, and 2.5 μg hCas9 plasmid (Addgene, 41815) for cell lines that did not harbor inducible Cas9 transgenes. Electroporation was performed with an exponential decay waveform, 250V, and mTESR1 medium, counted, and subsequently plated at a density of 2000 cells/cm^2 on ArrayEdit in mTESR1 medium (WiCell) containing 10μM ROCK inhibitor (Y27632, Selleckchem). Cells remained undisturbed and allowed to attach for 48 hours followed by daily media changes.

Human embryonic kidney cells were plated at 50,000 cells/well in 24 well plate (~25,000 cells/cm^2) at day 0. On day 1, 50 µL of Transfection media was added to cells in 450 µL of freshly exchanged Growth media. Transfection media contained 50 µL Opti-MEM (Life Technologies), 1.25 µg Cas9 plasmid, and 0.75 µL of Lipofectamine 2000 (Life Technologies). On day 2 50 µL of Transfection media was added to cells in 450 µL of freshly exchanged Growth media. Transfection media contained 100 µL Opti-MEM (Life Technologies), 0.75 µL of Lipofectamine 2000 (Life Technologies), and either 1.25 µg one-pot sgRNA. 1.25 µg plasmid-based IVT, 0.5 µg sgRNA plasmid, or 3 nM U6-gBlock [Integrated DNA Technologies
(Prediger, 2015)]. Cells were cultured in Growth media plus Transfection media for 4 additional days before downstream analysis.

Embryoid body differentiation

Cells were cultured created using the Aggrewell (Stem Cell Technologies) system per the manufacturer's protocol. EBs were cultured in non-adherent cell culture plates (Corning Inc.) suspended in 4 mL per well of "EB Medium" consisting of 80% Iscove's Modified Dulbecco's Medium (IMDM, Life Technologies) with 20% Fetal Bovine Serum (Life Technologies). After four days in suspension, RNA was harvested from aggregates using SV Total RNA Isolation System (Promega) according to manufacturer instructions.

Deep sequencing library preparation

RNA was reverse transcribed using SuperScript III (Life Technologies). 30 ng of each RNA extraction were combined with random hexamer primers and water before incubating for 5 minutes at 65 ºC. SuperScript III enzyme mix was then added and the reaction was thermocycled for 5 minutes at 25 ºC , 50 minutes at 50 ºC followed by termination of the reaction at 85 ºC for 5 minutes. DNA was then amplified using gene specific primers with Illumina adapters using Taq AccuPrime HiFi polymerase as described in Genomic Analysis. Genomic PCRs were then purified using Wizard SV Gel and PCR Clean-Up System (Promega) and quantified using a Nanodrop2000. Samples were then pooled and ran on an Illumina HiSeq 2000 at a run length of 1x100bp.

Deep sequencing data analysis

A custom python script was developed to perform sequence analysis. The pipeline starts with preprocessing, which consists of filtering out low quality sequences and finding the defined ends of the reads. For each sample, sequences with frequency of less than 100 were filtered from the data. Sequences in which the reads matched with primer and reverse complement subsequences classified as "target sequences". Target sequences were aligned with corresponding wildtype sequence using global pairwise sequence alignment from "Biopython" package (http://biopython.org). Each aligned sequence was classified as follows. If there is no insertion or deletion in the aligned sequence, it classified as the wild type otherwise it considered a mutant. If the mutant sequence preserves the reading frame, the sequence is called in-frame mutant; otherwise, it is a frame-shift mutant. This sequencing assay would not lose information if a premature STOP codon is produced by the editing, but rather can detect these edited transcripts. The frequency, length and position of matches, insertions, deletions and mismatches were identified in the resulting aligned sequences. The frequency and position of in-frame shifts were also extracted and compared in different samples. The custom python script is available upon request.

Microcontact-printed (*μ*CP) well plate fabrication

*μ*CP was performed using previously described methods (Harkness et al., 2015). The surface modification involved printing of an alkanethiol initiator to nucleate the polymerization of hydrophilic poly(ethylene glycol) (PEG) chains (**Fig. S2A**). Briefly, double sided-adhesive was attached to the bottom of a standard tissue culture plate, after which a laser cutter was used to cut out the well bottoms. Glass sheets were purchased at a size slightly smaller than a well plate. A metal evaporator was then used to deposit a thin layer of titanium, followed by a layer of gold onto one side of the glass sheet. Using previously described chemistry (Sha et al., 2013), patterns were transferred to gold-coated glass via a polydimethylsiloxane stamp after which the glass was submerged in a poly(ethylene glycol) (PEG) solution overnight to build hydrophillic PEG chains surrounding *μ*Features. After submersion, sheets were washed with deionized water to remove residual copper deposited by the reaction and 70% ethanol to sterilize. Standard tissue culture plates with well bottoms cut out were then fastened to processed sheets using a custom-made alignment device.

Genomic analysis

DNA was isolated from cells using QuickExtract (Epicentre) following treatment by 0.05% trypsin-EDTA and centrifugation. QuickExtract solution was incubated at 65ºC for 15 minutes, 68ºC for 15 minutes, and finally 98ºC for 10 minutes. Genomic PCR was performed following manufacturer's instructions using AccuPrime HiFi Taq (Life Technologies) and 500 ng of genomic DNA. Products were then prepared for sequencing by purifying the PCR product using the QIAquick PCR purification kit (Qiagen) and quantified using a Nanodrop2000. PCR amplicons were submitted to the UW Biotechnology Center for Sanger Sequencing, and the sequencing chromatograms were analyzed in Benchling. For the *LAMA5* sgRNAs used in this study, all off-target sites scoring 1.0 and greater on a 100 point scale were assayed. The remaining sites dropped off in score significantly and are predicted to be modified at even lower probabilities.

Flow cytometry

Flow cytometry was performed using a C6 Accuri (BD) and analyzed using BD CSampler Software. mCherry fluorescence was detected using the 610/20 filter in the FL3 position. Gates were established by running wild type

WA09 hESCs. Data in Figure 1 are for two independent experimental replicates (n=2). For the Plasmid+Sort experiments, hESCs were co-electroporated with a GFP plasmid (Addgene #6085-1) in addition to plasmids encoding sgRNAs and Cas9, and then sorted for GFP+ cells using an Aria III sorter (BD Biosciences) 1 day after electroporation. The level of apoptosis was detected by trypsinization followed by fixation in 4% paraformaldehyde (IBI Scientific) at room temperature for 15 min. Cells were resuspended with 0.5% Triton-X 100 (Electron Microscopy Sciences) and incubated at room temperature for 30 min. Samples were then resuspended in 5% goat serum and a primary antibody against cleaved caspase-3 (1:400 Cell Signaling Technologies 9661S) and placed at 4 ºC overnight. The next day, cells were centrifuged and resuspended in 5% goat serum plus a goat anti-rabbit secondary antibody conjugated to AlexaFluor488 (1:400 Santa Cruz Biotechnology sc-362262). Gates were established by running a secondary antibody only control.

Immunocytochemistry
Biallelic-edited hESC clones were cultured on matrigel for >10 passages and then fixed in 4% paraformaldehyde (IBI Scientific) at room temperature for 15 min. Cells were washed with PBS then permeablized with 0.5% Triton-X 100 (Electron Microscopy Sciences) by incubation at room temperature for 30 min. Primary antibodies against Oct4 conjugated to AlexaFluor594 (1:10 BD Pharmingen 560186), Nanog (1:200 R&D Systems AF1997), and TRA-1-60 (1:100, EMD Millipore MAB4360) were prepared in 5% donkey serum and incubated overnight at 4ºC. For primary antibodies without conjugated antibodies, samples were washed with 5% donkey serum then incubated in PerCP/Cy5.5 (406511) or AlexaFluor-647 (ThermoFisher A21238) anti-mouse or goat secondary antibodies diluted 1:400 in 5% goat serum at 4ºC for 1 hour. To detect apoptosis, a primary antibody against cleaved caspase-3 (1:250 Cell Signaling Technologies 9661S) was used as described above and detected using anti-rabbit, AlexaFluor-488 conjugated secondary antibody (ThermoFisher A11034). Cells were analyzed using fluorescent microscopy with a Nikon TiE microscope as described in the high-content imaging section.

Statistical analysis
All statistical tests were performed using Excel standard packages (Microsoft). Student's t-tests were performed using two-tailed, two-sample unpaired data with unequal variance and were deemed significant at $p<0.05$.

SUPPLEMENTAL REFERENCES

Chen, G., Gulbranson, D.R., Hou, Z., Bolin, J.M., Ruotti, V., Probasco, M.D., Smuga-Otto, K., Howden, S.E., Diol, N.R., Propson, N.E., Wagner, R., Lee, G.O., Antosiewicz-Bourget, J., Teng, J.M.C., Thomson, J.A., 2011. Chemically defined conditions for human iPSC derivation and culture. Nat. Methods 8, 424–429. doi:10.1038/nmeth.1593

Prediger, E., 2015. Simplifying CRISPR. Genet. Eng. Biotechnol. News 35, 18–19. doi:10.1089/gen.35.12.11

SUPPLEMENTAL TEXT

Below is an example of a code used in MySQL Workbench for implementation of ArrayEdit. Comments are preceded by a # and in grey.

```
#select all tables from each day of experiment, Image_Frame tables contain data about each image in series

SELECT Day6.Image_Frame_TxRed as Frame, Day2.Image_Count_H9_H2B as Day2Count,
Day3.Image_Count_H9_H2B as Day3Count, Day4.Image_Count_H9_H2B as Day4Count,
Day5.Image_Count_H9_H2B as Day5Count, Day6.Image_Count_H9_H2B as Day6Count

FROM cellprofilerdb1.JCS2_5_LamA5KOPer_Image Day2


#join together each day on the basis of the frame. Frame is consistent from day today as it conveys spatial
information within the well. Uses one day as master table to join other tables to, decreasing the likelihood of error

join cellprofilerdb1.JCS2_5_LamA5KOPer_Image Day3 on Day2.Image_Frame_TxRed=Day3.Image_Frame_TxRed

join cellprofilerdb1.JCS2_5_LamA5KOPer_Image Day4 on Day2.Image_Frame_TxRed=Day4.Image_Frame_TxRed

join cellprofilerdb1.JCS2_5_LamA5KOPer_Image Day5 on Day2.Image_Frame_TxRed=Day5.Image_Frame_TxRed

join cellprofilerdb1.JCS2_5_LamA5KOPer_Image Day6 on Day2.Image_Frame_TxRed=Day6.Image_Frame_TxRed

#when using 6-well plates, each well must be imaged individually and is named by the position of the well.  This
check ensures that only like wells will be joined together across days. Wildcards are used in case a day was
misnamed with extra spaces or trailing characters.  Standard notation is DayX-WellXX.

where Day2.Image_FileName_TxRed like '%2-WellB2%' and Day3.Image_FileName_TxRed like '%3-WellB2%' and
Day4.Image_FileName_TxRed like '%4-WellB2%' and Day5.Image_FileName_TxRed like '%5-WellB2%'and
Day6.Image_FileName_TxRed like '%6-WellB2%'


#Discard any wells that are not of interested of may produce misleading data due to the presence of 0's.

and Day2.Image_Count_H9_H2B != 0 and Day3.Image_Count_H9_H2B!=0 and Day4.Image_Count_H9_H2B!=0
and Day5.Image_Count_H9_H2B!=0 and Day6.Image_Count_H9_H2B!=0

order by Day2.Image_Frame_TxRed;
```
